# The nuclear and mitochondrial genomes of *Frieseomelitta varia* – a highly eusocial stingless bee (Meliponini) with a permanently sterile worker caste

**DOI:** 10.1186/s12864-020-06784-8

**Published:** 2020-06-03

**Authors:** Flávia C. de Paula Freitas, Anete P. Lourenço, Francis M. F. Nunes, Alexandre R. Paschoal, Fabiano C. P. Abreu, Fábio O. Barbin, Luana Bataglia, Carlos A. M. Cardoso-Júnior, Mário S. Cervoni, Saura R. Silva, Fernanda Dalarmi, Marco A. Del Lama, Thiago S. Depintor, Kátia M. Ferreira, Paula S. Gória, Michael C. Jaskot, Denyse C. Lago, Danielle Luna-Lucena, Livia M. Moda, Leonardo Nascimento, Matheus Pedrino, Franciene Rabiço Oliveira, Fernanda C. Sanches, Douglas E. Santos, Carolina G. Santos, Joseana Vieira, Angel R. Barchuk, Klaus Hartfelder, Zilá L. P. Simões, Márcia M. G. Bitondi, Daniel G. Pinheiro

**Affiliations:** 1 Departamento de Genética, Faculdade de Medicina de Ribeirão Preto, Universidade de São Paulo, Ribeirão Preto, SP Brazil; 2 Departamento de Biologia Celular e do Desenvolvimento, Instituto de Ciências Biomédicas, Universidade Federal de Alfenas, Alfenas, MG Brazil; 3 Departamento de Ciências Biológicas, Faculdade de Ciências Biológicas e da Saúde, Universidade Federal dos Vales do Jequitinhonha e Mucuri, Diamantina, MG Brazil; 4 Departamento de Genética e Evolução, Centro de Ciências Biológicas e da Saúde, Universidade Federal de São Carlos, São Carlos, SP Brazil; 5 Universidade Tecnológica Federal do Paraná, Cornélio Procópio, PR Brazil; 6 Departamento de Biologia Celular e Molecular e Bioagentes Patogênicos, Faculdade de Medicina de Ribeirão Preto, Universidade de São Paulo, Av. Bandeirantes 3900, Ribeirão Preto, SP 14049-900 Brazil; 7 Departamento de Tecnologia, Faculdade de Ciências Agrárias e Veterinárias, Universidade Estadual Paulista “Júlio de Mesquita Filho”, Jaboticabal, SP Brazil; 8 Departamento de Biologia, Faculdade de Filosofia, Ciências e Letras de Ribeirão Preto, Universidade de São Paulo, Ribeirão Preto, SP Brazil

**Keywords:** Social insect, Meliponini, Illumina sequencing, Genome assembly, Synteny, Repetitive elements, Non-coding RNA, Reproductive process genes, Immunity genes

## Abstract

**Background:**

Most of our understanding on the social behavior and genomics of bees and other social insects is centered on the Western honey bee, *Apis mellifera.* The genus *Apis,* however, is a highly derived branch comprising less than a dozen species, four of which genomically characterized. In contrast, for the equally highly eusocial, yet taxonomically and biologically more diverse Meliponini, a full genome sequence was so far available for a single *Melipona* species only. We present here the genome sequence of *Frieseomelitta varia*, a stingless bee that has, as a peculiarity, a completely sterile worker caste.

**Results:**

The assembly of 243,974,526 high quality Illumina reads resulted in a predicted assembled genome size of 275 Mb composed of 2173 scaffolds. A BUSCO analysis for the 10,526 predicted genes showed that these represent 96.6% of the expected hymenopteran orthologs. We also predicted 169,371 repetitive genomic components, 2083 putative transposable elements, and 1946 genes for non-coding RNAs, largely long non-coding RNAs. The mitochondrial genome comprises 15,144 bp, encoding 13 proteins, 22 tRNAs and 2 rRNAs. We observed considerable rearrangement in the mitochondrial gene order compared to other bees. For an in-depth analysis of genes related to social biology, we manually checked the annotations for 533 automatically predicted gene models, including 127 genes related to reproductive processes, 104 to development, and 174 immunity-related genes. We also performed specific searches for genes containing transcription factor domains and genes related to neurogenesis and chemosensory communication.

**Conclusions:**

The total genome size for *F. varia* is similar to the sequenced genomes of other bees. Using specific prediction methods, we identified a large number of repetitive genome components and long non-coding RNAs, which could provide the molecular basis for gene regulatory plasticity, including worker reproduction. The remarkable reshuffling in gene order in the mitochondrial genome suggests that stingless bees may be a hotspot for mtDNA evolution. Hence, while being just the second stingless bee genome sequenced, we expect that subsequent targeting of a selected set of species from this diverse clade of highly eusocial bees will reveal relevant evolutionary signals and trends related to eusociality in these important pollinators.

## Background

The ecological and economic importance of bees as pollinators and their millennial association with man, especially of the highly eusocial honey bees (Apini) and stingless bees (Meliponini) as providers of honey, pollen, wax, and propolis, has, not surprisingly, been a key factor for including the Western honey bee *Apis mellifera* in a top priority position for genome sequencing at the beginning of this century. In fact, the honey bee nuclear genome was the third insect genome to be sequenced [1], and is now one of the best annotated ones with over 15,000 predicted protein-coding genes [2, 3]. As such, it generally serves as a major backbone for sequencing and annotation efforts of other genomes, especially so within the Hymenoptera, the phylogenetically most ancient branch within the holometabolous insects [4].

*Apis mellifera* is a model organism for understanding social organization, especially so the permanent caste systems of highly eusocial insects. Nonetheless it is actually a member of the smallest branch within the monophyletic clade of corbiculate bees [5, 6] that comprise the highly eusocial Apini and Meliponini [7], as well as the primitively eusocial bumble bees (Bombini) and the solitary to incipiently social orchid bees (Euglossini). The tribe Apini comprises a single genus, *Apis*, of less than a dozen species, and for four of these fully sequenced genomes are available (*A. mellifera* [1, 2], *A. florea* [8], *A. cerana* [9], and *A. dorsata* [10].

This stands in strong contrast with the stingless bees (Meliponini), which comprise over 500 species classified into 48–61 genera [6, 11]. The largest number of genera and species occurs in the Neotropics, with 32 genera and 417 recognized species [12], and recent population genetics studies indicate that species numbers are likely to be even higher [13]. Nonetheless, only one of these stingless bee species, *Melipona quadrifasciata*, has a fully sequenced and annotated genome, as it was included in a comparative genomics study of bees aimed at providing insights into genomic traces of social evolution [8]. A second species, *Lepidotrigona ventralis*, a Southeast Asian species recently had raw genome sequence data deposited in GenBank [PRJNA387986], but genomic annotation is still lacking.

Stingless bees are not only a species-rich monophyetic clade, they are also phylogenetically much older than the Apini, with origins dating back to 75–80 million years ago (mya) [11], compared to the origin of Apini, which is set at 22 mya. The Gondwana origin of the Meliponini can be seen reflected in the vicariance of their biogeographical, pantropical distribution [11].

In the tropical and subtropical Americas, the stingless bees were the main pollinators until the introduction of the honey bee, *A. mellifera*, in the eighteenth century. They have higher population densities than the solitary or primitively eusocial bees, and they are generalist plant visitors [14], which makes them also ideal pollinators for economically valuable crops, including greenhouse crops. The management of stingless bees (meliponiculture) has a long history, as shown in Pre-Colombian documents, such as the Maya Codex Madrid, that records practices for *Melipona beechei* from Mesoamerica. Also, over the last decades, meliponiculture has gained new momentum as part of subsistence agriculture [15].

Stingless bees are also highly varied in important biological aspects, including colony size, nesting sites, communication systems, and colony defense, as well as caste determination and reproductive biology. For instance, while colonies of the tiny, fruit fly-size *Leurotrigona* species can fit into a matchbox, colonies of the open-nesting *Trigona* species can be of a size comparable to that of very large honey bee colonies. In terms of nesting sites, most stingless bees are cavity nesters, mostly so in trees, but they can use pretty much any kind of cavity, including underground ones [16, 17].

With respect to caste determination, the genus *Melipona* has long drawn attention, as it was the first social insect species for which a genetic mechanism of queen/worker determination was proposed [18], with underlying mechanisms still under investigation [19–21]. Nonetheless, it is in their reproductive biology in general that the stingless bees differ most drastically from the honey bees, and in this respect they are actually much closer to the bumble bees, with which they have a sister group relationship [5, 6]. The queens of most stingless bees mate with a single male only, and in many species, the workers contribute to the production of males in a colony [22]. In contrast, in the genera *Frieseomelitta* and *Leurotrigona*, the workers are completely sterile, and for *Frieseomelitta varia* it has been shown that the ovaries of workers undergo complete programmed cell death during pupal development [23].

In the previous comparative genomics study on sociality in bees [8], *M. quadrifasciata* was included not only for being the first among the stingless bees to have its genome sequenced, but also because of its emblematic genetic mode of caste determination. Furthermore, *M. quadrifasciata* and *F. varia*, are the two only stingless bee species for which RNA-Seq data had previously been generated in a comparative transcriptomics study [24]. Hence, we chose here the species *Frieseomelitta varia* for genome sequencing of a third candidate of this largest clade of highly eusocial bees, the Meliponini.

## Methods

### Sampling and DNA extraction

Brood cells were removed from *F. varia* colonies kept in the apiary of the Department of Genetics, Ribeirão Preto Medical School, University of São Paulo, and screened for the presence of male brood. The use of haploid males, as done in previous bee genome projects [1, 8], presents a considerable advantage for genome assembly. Thus, we also opted to use whole body DNA from a single late pupal-stage male specimen with still unpigmented wings. A voucher specimen of the respective colony was deposited in the Entomological Collection RPSP (Coleção Entomológica Prof. J.M.F. Camargo, FFCLRP/USP) under the register USP_RPSP 00005682. Genomic DNA was extracted using the Wizard® Genomic DNA Purification Kit (Promega, Madison, WI) resulting in a sample of 9.7 μg total DNA.

### Genomic DNA library preparation and sequencing

The DNA sample was sent to *Laboratório Central de Tecnologias de Alto Desempenho em Ciências da Vida* (LaCTAD, UNICAMP, Campinas, Brazil) for quality check (2100 Bioanalyzer, Agilent Technologies, Santa Clara, CA), library preparation, and sequencing. Library preparation was done using Illumina Nextera kits (Illumina, San Diego, CA), and paired-end and mate pair sequencing was done on a HiSeq 2500 platform (Illumina). The extracted DNA was used for the construction of three sequencing libraries: two paired-end (one lane each) and one mate-pair (one lane). The paired-end libraries were prepared according to the TruSeq™ DNA Nano Library Preparation Protocol (Illumina) using 100 ng input DNA. After DNA shearing, 350 bp inserts were selected using a bead-based method. Inserts were amplified by 8 PCR cycles, and the sequencing reaction yielded 2 × 101 bp reads. The mate-pair library was prepared from 1 μg of input DNA, following the Nextera® Mate Pair Library Preparation Protocol (Illumina). Fragments of 3 kb were circularized and sheared followed by purification of mate-pair fragments using beads. Mate-pair fragments were amplified in 10 PCR cycles, and the sequencing reaction produced 2 × 101 bp reads.

### Genome assembly

Raw reads were submitted to quality analysis using FastQC software (www.bioinformatics.babraham.ac.uk/projects/fastqc/). The paired-end reads were analyzed with Trimmomatic software [25] v. 0.35, which carried out the following tasks: removal of TruSeq DNA 3′ adapters (ILLUMINACLIP:TruSeq3- PE.fa:2:30:10); removal of leading low quality or N bases (below quality 3) (LEADING:3); removal of trailing low quality or N bases (below quality 3) (TRAILING:3); scanning of the reads with a 4-base wide sliding window, and cutting when the average quality per base drops below 15(SLIDINGWINDOW:4:15); dropping reads < 100 bases (MINLEN:100). The mate-pair reads were analyzed using NxTrim [26] v. 0.4.1 to discard low quality reads and categorize reads according to the orientation implied by the adapter location. Thus, NxTrim builds “virtual libraries” of mate pairs, paired-end reads and single-ended reads, and, also trims off adapter read-through. NxTrim was executed with an aggressive adapter search (−-aggressive parameter) to retrieve only genuine mate-pair reads.

An initial assembly was obtained using SPAdes software v. 3.9.0 [27], with error correction module (BayesHammer) enabled and using multiple k-mer sizes (33–81 bp). This assembly was subsequently used as reference for read alignments using HISAT2 software v. 2.0.5 [28], considering end-to-end alignments, avoiding spliced alignments and setting the right range of insertion size for paired-end (100–600 bp) and mate-pair (1–15 Kbp) libraries. The read alignments were used by BESST software v. 2.2.4 [29] to scaffold the initial genome assembly, considering only the alignments with mapping quality greater than or equal to 30. The scaffold size distribution was calculated by using functions implemented in R Statistical Software [30]. The assembly version thus generated was named Fvar-1.2.

Genome heterozygosity, repeat content, and size were evaluated from sequencing reads by a k-mer-based statistical approach. This was done in GenomeScope software v. 1.0, using as input the histogram file from k-mer frequency counting generated in the software Jellyfish v.2.2.0 (k-mers 19–63). A genome evaluation analysis of this assembly was made using QUAST-LG v. 5.0.2. Details on softwares and scripts are available in: https://github.com/dgpinheiro/fvaria/.

### Prediction and annotation of protein coding genes

Initial gene predictions were made using MAKER2 software [31] version 2.31.8 in conjunction with UniProt sequences for *A. mellifera*. These automated predictions were further refined by using transcriptome data from *F. varia* RNA-Seq libraries generated from abdominal and brain RNA of adult workers [24] (SRA accession number SRR098304), as well as predictions for protein coding genes of *A. mellifera* (GCF_000002195.4). Additional gene model support came from RNA-Seq libraries generated for integument RNA from preimaginal and adult stages [32]. The raw sequences of those RNA-Seq libraries (NCBI BioProject ID PRJNA490324) were assembled in Trinity software with score definitions provided by the DETONATE tool [33] and used for alignment with the Fvar-1.1 genome assembly using MAKER2.

BUSCO software v.2.0 [34] was used to evaluate the completeness of the *F. varia* gene annotation. We conducted the BUSCO analysis of single-copy orthologs using the Hymenoptera datset (OBP9) considering the predicted transcriptome extracted from the *F. varia* genome. For the annotation of coding gene functions we used the eggNOG-mapper v. 2.0.0 software [35] and generated a fasta and GFF file for the protein sequences (Fvar-1.2-proteins.fa; Fvar-1.2.gff).

For further validation of the predicted gene models, a set of 533 genes was selected for manual curation. As a first step, the honey bee orthologs for these genes were used as queries in blastp searches against the respective protein predictions for *F. varia* [Fvar-1.2-proteins]*,* and the prospective orthologs were mapped against the assembled *F. varia* genome using the ARTEMIS platform [36] (https://www.sanger.ac.uk/science/tools/artemis). Exon and splice site predictions, as well as position of the automatically predicted gene models were checked, and in the case of genes that were split across scaffolds this was recorded. For each manually curated gene a GFF file was created, which contained information on problems and suggestions for gene model correction.

The identification of basal and other transcription factor (TF) domains in the predicted proteins of *F. varia* followed an approach similar to that taken by Kapheim et al. [8]. The function “hmmscan” of HMMER 3.1b2 [37] was used to scan the protein sequences (option --cut_ga and E-value ≤1e^− 5^) against the Pfam-A database [38] (Pfam-A.hmm from ftp://ftp.ebi.ac.uk/pub/databases/Pfam). The results were filtered for a curated list of TFs retrieved from the Transcription factor database v2.0 [39] (www.transcriptionfactor.org). Other TF domains and basal TF domains were retrieved from Huang et al. [40] and included in the analysis. We calculated the proportion of genes with basal and other TFs. For this, we divided the number of genes with TF domains by the total number of predicted genes, and then compared this number to the proportion of genes with TF domains reported for the 10 previously published bee species [8].

### Gene Ontology analysis

For the predicted genes, a functional analysis was performed using Blast2GO software, version 4 (https://www.blast2go.com/), with the following steps: (1) a blastp search against the GenBank NR database, (2) an InterproScan sequence search (https://www.ebi.ac.uk/interpro/search/sequence-search), (3) mapping to retrieve Gene Ontology (GO) terms to the sequence, and (4) attributing the EC code to the respective proteins.

### Synteny analysis

To check for synteny in the gene organization of *F. varia* with known linkage groups in *A. mellifera,* the respective orthologs were first identified by means of reciprocal blastp searches. Their genomic localization in the honey bee genome (version 4.5) was retrieved and mapped against the coordinates of the *F. varia* genome assembly version 1.1 using scripts written in Python (https://www.pythons.org). Synteny was plotted using Circos software package [41] for visualizing genomic data.

### Phylogenetic analysis of gene families and functional groups

Gene families and functional groups selected for manual curation were analyzed with respect to their phylogenetic relationships among bees. For this, their orthologs were retrieved by blastp searches against the sequences of 13 published bee genomes (Table S1) using the Hymenopteramine tool (http://hymenopteragenome.org/hymenopteramine/begin.do) available in Hymenopterabase [2]. For each gene family or functional group of interest a FASTA file containing the respective amino acid sequences of the 14 bee species (*F. varia* + 13) was generated and the sequences were aligned using the MAFFT program v7.402 with the L-INS-i approach [42] (https://mafft.cbrc.jp/alignment/software/). Gene phylogenetic trees were reconstructed by means of the Randomized Axelerated Maximum Likelihood (RAxML) program version 8.2.10 [43] (https://cme.h-its.org/exelixis/web/software/raxml/), with the CAT model, the JTT substitution matrix, and 1000 bootstrap replications. All programs are implemented in the CIPRES platform [44]. The trees were edited using FigTree v1.4.3 (http://tree.bio.ed.ac.uk/software/figtree/).

### Prediction and analysis of non-protein coding genes and repetitive genomic components

Non-coding RNA (ncRNAs) genes were predicted using sequence similarity and structural search strategies. BLAST tools were used to search against ncRNAs Ensembl Insects (*Anopheles gambiae* AgamP4, *Apis mellifera* Amel_4.5, *Atta cephalotes* Attacep1.0, *Bombus impatiens* BIMP_2.0, *Bombyx mori* ASM15162v1, *Drosophila melanogaster* BDGP6, *Nasonia vitripennis* Nvit_2.1 and *Solenopsis invicta* Si_gnG) and InsectBase databases (piRNA and lncRNA).

For the identification of microRNA genes, BLAST searches were performed against miRBase release 22.1 [45]. For structural ncRNA gene searches, we used INFERNAL version 1.1.2 [46] based on the Rfam 14.0 database [47]. The INFERNAL annotation used CMsearch software with the parameter –cut_ga. The BLAST search used filter dust, and an E-value = 0.00001 and identity/coverage of at least 95% as thresholds. All filtering and merge results steps were performed by customized Perl scripts. The insect species and respective genome versions used are listed in Table S2.

For a more detailed analysis of the microRNA gene *miR-34* the corresponding sequences in arthropods were retrieved from miRBase version 21 [44] and aligned in CLUSTALW [48, 49] and analyzed in MEGA v.7 [50] using the Maximal Parsimony method and 100 bootstrap replications.

For the identification of long non-coding RNA (lncRNA) genes we first extracted intergenic regions based on coding annotation from the *F. varia* genome. Intergenic sequences longer than 200 nucleotides retrieved using a Perl script from the RNAplonc tool (200 nt.pl) [51] were considered for further analysis. Protein encoding potential was filtered out by using the CPAT tool [52] with ORF_size ≥100 and Coding Prob. ≥0.345, and the remaining candidate lncRNA gene sequences were analyzed against Pfam FASTA data [38] using the blastx tool with an E-value of 10^− 5^ and SEGfilter for low complexity regions. All ‘no-hit’ sequences were then considered as lncRNA genes. Finally, we compared the lncRNA annotations with non-coding RNA annotations to discard lncRNA candidates that might overlap with non-coding RNA annotations, so that only *bona fide *lncRNA candidates would be kept in the list.

For the identification of repetitive elements we used RepeatMasker tool v4.1.0 [53] (default parameters) with RepBase version database RepBase26.10.2018, and RepeatModeler version 2.0.1. We merged both results by the overlapping of genomic regions to avoid redundancy results by using a Perl script.

### Mitochondrial genome analysis

#### Assembly

Four different softwares, NOVOPlasty v.2.59 [54], SPAdes v. 3.6.2 [27], Platanus v. 1.2.4 [55] and MitoBim v. 1.9 [56] were employed. Using all trimmed paired-end reads, as recommended, with 39 k-mers, and the COX2 sequence of *Bombus hypocrita sapporensis* (NC_011923) as seed, the organelle-specific software NOVOPlasty turned out to be the only procedure that generated a unique, single contig with evidence for circularization. The other softwares also generated assemblies with the same gene content, but these were still fragmented.

#### Validation

The first validation step was to assess the correspondence and coverage of the reads against the assembled mitochondrial genome. For this, only high-quality paired-end reads were selected and processed using PrinSeq v.2.20.3, under the following parameters: trimming was done using a sliding window approach, considering a quality score mean < 28 in a window size of 3 bp sliding 1 bp to the left, in addition to filtering sequences with at least one quality score < 30. The paired-end reads were aligned against the assembled mitochondrial genome using Bowtie v. 0.12.7 (with the parameters: end-to-end hits with up to 1 mismatch, and with a maximum insert size of 300 for paired-end). Usinge the same mapped reads, we used the program REAPR v.1.0.18 [57], which does not require a reference genome, for evaluation of reads coverage, counts of unique mappings, and absence of mismatches.

An additional validation approach was performed using the alignment of paired-end reads. With this we checked if there were alignments of fragments across block junctions, i.e., whether the two reads of each pair aligned in each adjacent block, thus supporting adjacency. First, with the software MAUVE v. 2.4.0 [58], we performed a multiple genome alignment for information on genome synteny between mitochondrial genome regions of the *F. varia* assembled genome with those of *Apis mellifera* (NC001566), *Bombus hypocrita* (NC011923) and *Melipona scutellaris* (NC026198) mitochondrial genomes [59]. From this alignment, we defined six genome blocks in the assembled genome, according to rearrangements observed in the mitochondrial gene order compared to other high eusocial bee species. With these blocks we obtained the respective coordinates of the rearrangements and used them as input files in specifically developed in-house scripts (https://github.com/dgpinheiro/fvaria#assembly-of-mitochondrial-genome).

#### Annotation

This was initially done using the software MITOS2 (http://mitos2.bioinf.uni-leipzig.de/index.py). As this program does not correctly detect initiation and stop codons, it was necessary to posteriorly manually adjust for these using ORFfinder (https://www.ncbi.nlm.nih.gov/orffinder/) and BLAST tools, which were also used to identify rRNA coordinates. tRNAs were identified by means of the softwares tRNAscan-SE 2.0 [60] and ARWEN [61] using standard parameters. A map of the *F. varia* mitochondrial genome was produced using OGDRAW (OrganellarGenomeDRAW00) [62].

#### Phylogenetic analyses

A multiple genome alignment was generated for the comparison of the assembled *F. varia* mitochondrial genome with complete mitochondrial genomes of the superfamily Apoidea. Three concatenated genes datasets were separated: one with all mitochondrial genes (coding sequences + tRNAs + rRNAs), with only protein coding sequences, and the third one consisting only of the tRNAs. All datasets were aligned using the MAFFT v.7 webserver [63], with standard parameters. The analyses were done using Maximum Likelihood (ML) method in the RAxML softwares using Rapid bootstrap [64] and Bayesian Inference (BI) in Mr. Bayes [65]. The evolutionary models were calculated with jModelTest v.2 [66]. All softwares were run online at the CIPRES Science Gateway. For finding the ML tree, 10,000 replicates were used and clade consistency was evaluated by 1000 bootstrap replicates. For Bayesian Inference, two runs and four chains were calculated with 5,000,000 generations until reaching an average standard deviation of split frequencies of less than 0.01. The 25% of the initial trees were discarded as burn-in. The outgroup was represented by two ant species [*Anoplolepis gracilipes* (NC_039576) and *Camponotus atrox* (NC_029357)], and four bee species [*Megachile sculpturalis* (NC_028017), *Rediviva intermixta* (NC_030284), *Hylaeus dilatatus* (NC_026468), and *Colletes gigas* (NC_026218)]. All trees were edited with the program TreeGraph 2 [67] and iTOL (https:itol.embl.de). The evolutionary models for the trees were: GTR + G for the complete dataset and the protein coding genes, and TIM1+ G for the tRNAs. The species included in the phylogenetic analysis are all listed in Table S3.

## Results

### Whole genome assembly

After an initial quality check using FastQC, the 2*251,808,069 Illumina paired-end reads (101 bp, 64.85% bases ≥ Q30) and 171,026,322 mate-pair reads (92.10% bases ≥ Q30) were trimmed using Trimmomatic and Nxtrim, respectively. This pre-processing resulted in a total of 234,357,438 high-quality paired-end and 9,671,088.161 high-quality mate pair reads. The results for the first assembly (Fvar-1.0) generated with SPAdes software was still highly fragmented, with almost 10,000 scaffolds. This assembly was considerably improved using HISAT2 and the BESST package for scaffolding. These generated the second assembly named Fvar-1.2. Details on these assemblies are given in Table 1.

**Table 1:** General statistics for the first two *F. varia* genome assembly versions. The genome evaluation was made using QUAST-LG v. 5.0.2

Statistics without reference	Fvar-1.0	Fvar-1.2
# contigs (> = 0 bp)	9755	2173
Largest contig	603,324	2,258,834
Total length (> = 100,000 bp)	116,286,290	255,941,895
N50	83,201	470,005
N75	43,559	244,533
L50	946	174
L75	2087	379
Total genome length		275,412,029
GC (%)		36.72

**Fig. 1:**
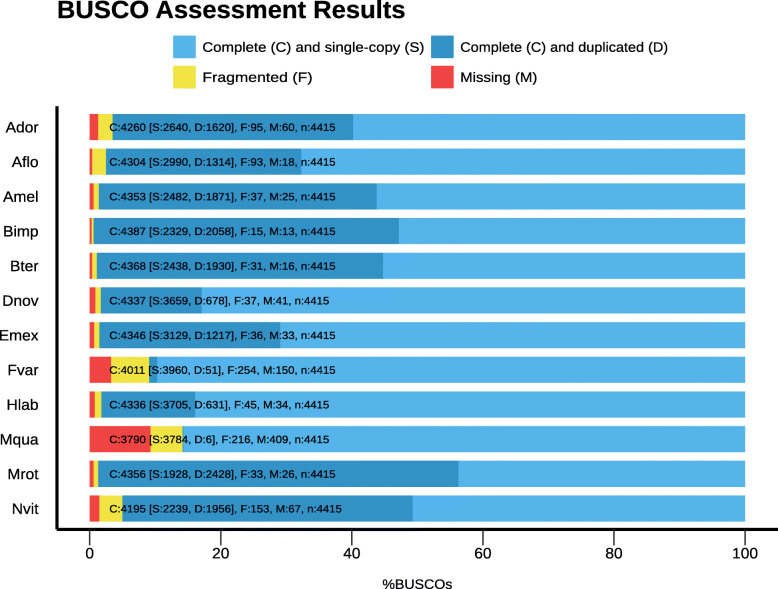
BUSCO analysis. The *F. varia* gene content was compared with that of 10 other bees and the parasitic wasp *Nasonia vitripennis* using the following databases: [Ador] *Apis dorsata* (GCF_000469605.1), [Aflo] *Apis florea* (GCF_000184785.2), [Amel] *Apis mellifera* (GCF_000002195.4), [Bimp] *Bombus impatiens* (GCF_000188095.1), [Bter] *Bombus terrestris* (GCF_000214255.1), [Dnov] *Dufourea novaeangliae* (GCF_001272555.1) [Emex] *Eufriesea mexicana* (GCF_001483705.1), [Fvar] *Frieseomelitta varia* (predicted transcriptome extracted from genome annotation of Fvar-1.2), [Hlab] *Habropoda laboriosa* (GCF_001263275.1), [Mqua] *Melipona quadrifasciata* (GCA_001276565.1), [Mrot] *Megachile rotundata* (GCF_000220905.1), and [Nvit] *Nasonia vitripennis* (GCF_000002325.3)

The total size estimate for the assembled genome was 275 Mbp, with a GC content of 37%, and our gene annotation approach resulted in a total of 10,526 protein-coding genes. Furthermore, in a BUSCO analysis for the 4415 hymenopteran ortholog genes (OBP9) we compared *F. varia* to 10 other bees [8, 68, 69] and the parastitic wasp *Nasonia vitripennis* (Fig. 1). With this, we identified 3970 complete and 298 fragmented genes (90 and 6.7%, respectively) as hymenopteran single-copy orthologs. Only 147 genes (3.3%) were not found in the current version of the *F. varia* genome. The proportion of single-copy orthologs is widely used to assess the quality of both genome assembly and gene annotation [70]. Thus, the identification of the majority hymenopteran genes validates our genome assembly and gene prediction approaches.

### Protein-coding genes

The BUSCO analysis indicated that the vast majority of hymenopteran genes is represented in the *F. varia* genome sequence. One way of refining the confidence of our data is by observing their functional coherence, and aiming at this we ran a Gene Ontology analysis, comparing information from *A. mellifera* and *F. varia* protein sets. For *A. mellifera* we used RefSeq-NCBI (release 103) containing 22,451 well-annotated and non-redundant proteins. For the 10,526 predicted gene models of *F. varia* it was possible to identify isoforms for approximately 5%, and therefore, the set of data used for GO analysis corresponded to 11,115 non-redundant predicted proteins. For both bees, 56% of the sequences in each protein set (12,629 in *A. mellifera* and 6276 in *F. varia*) were associated with at least one GO term. Such incompleteness in GO term assignment is common for non-model organisms, as is the case for most insects. Thus, the data were normalized based on the proportion of GO-annotated genes prior to performing further comparisons.

**Fig. 2:**
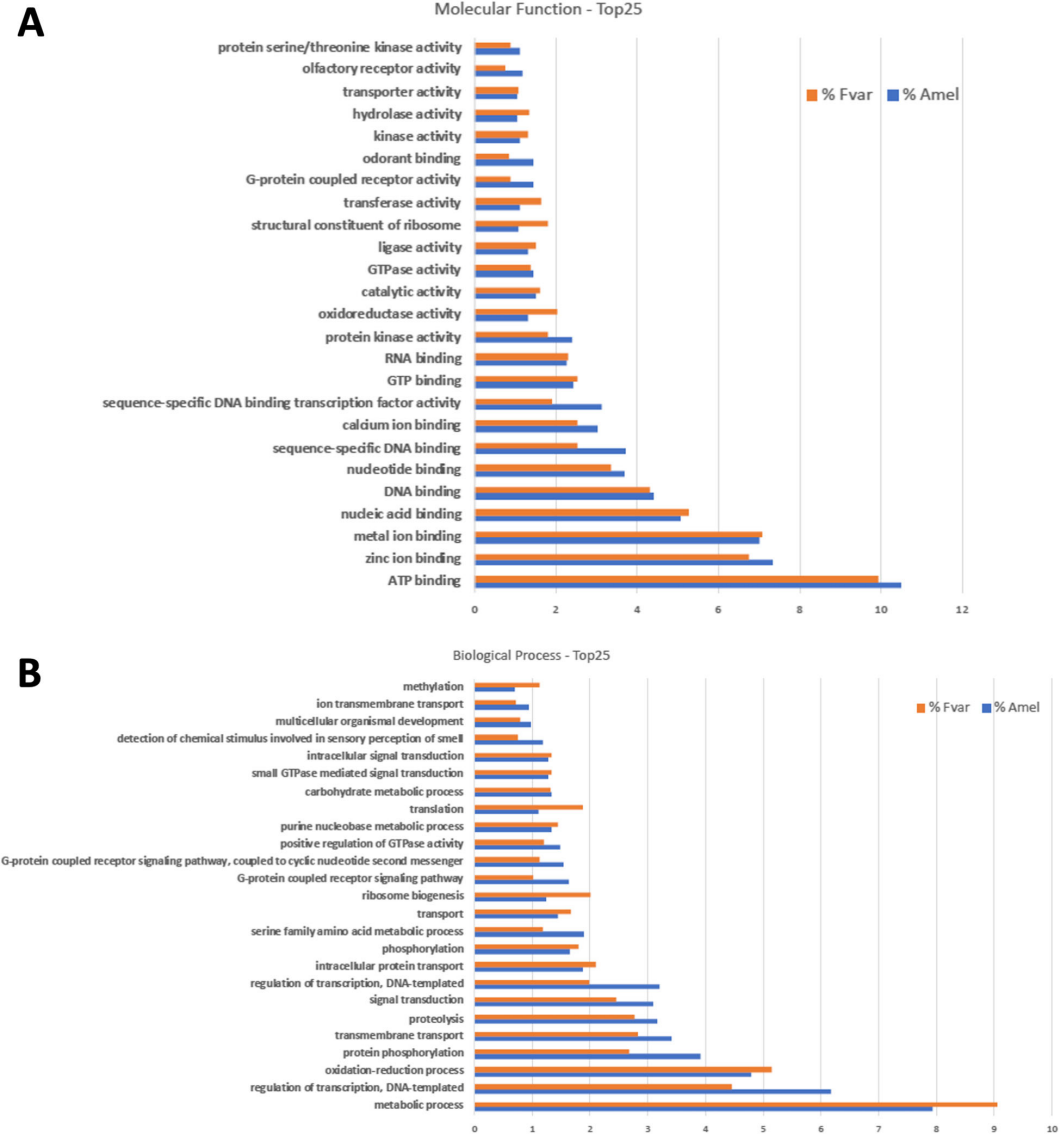
Frequency distribution of Gene Ontology categories in *Frieseomelitta varia* and *Apis mellifera*. **a** Top 25 categories for Molecular Function; **b** top 25 categories for Biological Process

Figure 2 reports the results for the top 25 Molecular Function and Biological Process categories comparing *F. varia* with *A. mellifera*. The percentages of genes with GO annotation for Biological Process and Molecular Function in the two species were similar in distribution profiles, indicating that any functional category, whether major, intermediate, or minor, is represented in approximately the same order of magnitude. This is in accordance with the view that the ontological-functional profiles are quite similar, even across large taxon borders [71].

Next, with the aim of evaluating the *F. varia* MAKER2 gene model predictions we selected 533 honey bee protein-coding genes as reference for the manual curation of their homologs in the *F. varia* genome using the ARTEMIS platform. These 533 genes were not chosen randomly from the honey bee OGS 3.2 set, but were included because of their functional association with developmental and reproductive processes, immunity, and processes related to social communication. These can, thus, be considered as of special interest for the social biology of stingless bees.

As a result of this manual curation of 533 genes, 241 (45,03%) of the automated predictions were considered 100% correct, but for the same percentage (45.02%, 240 genes), certain problems in exon assignment were noted, primarily for the first exon (Table S4). Furthermore, for 45 genes (8.3%) we could either not identify clear orthologs in the *F. varia* genome, or they were only found after manual searches. The remaining corrections (1.65%) were generally attributed to probable minor sequencing errors. A possible explanation for the misprediction of the first exon is a positional bias in the prediction of gene structure, in which an initial exon is less accurately identified compared to internal exons [72]. Especially, long first introns and longer introns in general [73], characteristics of higher eukaryotes genomes, impose extra challenge to accurately predict gene structures. Nonetheless, the overall quality of our genome assembly and gene annotation is confirmed by the high percentage of hymenopteran genes identified in the BUSCO analysis.

### Non-coding RNA genes

For a curated annotation of ncRNA classes in the *F. varia* genome we employed a combination of similarity-based and structure-based computational approaches, and we identified a total of 1946 ncRNAs (Table 2) falling into six ncRNA classes: small nuclear RNAs (snRNAs), small nucleolar RNAs (snoRNAs), transfer RNAs (tRNAs), ribosomal RNAs (rRNAs), intergenic long non-coding RNAs (lncRNAs), and microRNA precursors (miRNAs).

**Table 2:** Types and number of non-coding RNAs in the *F. varia* genome

ncRNA type	number	average length (bp)	total length (bp)
miRNA	103	89.81	9437
rRNA	21	339.8	7136
snoRNA	9	105.33	948
snRNA	53	150.43	7973
tRNA	180	74.08	13,335
lncRNA	1580	687.25	1,087,232
Total	1946		

Among the latter we identified members belonging to 38 miRNA families. We analyzed in more detail the microRNA-34, which is highly conserved in the animal kingdom and is maternally inherited in *D. melanogaster* [74] and in *A. mellifera* [75, 76], regulating the expression of important developmental genes. Its conservation was confirmed for *F. varia* and its sequence was seen to cluster closely with the honey bee (Figure S1).

Furthermore, we performed a comparative analysis on the distribution of ncRNA families in insect genomes of different orders. The results shown in Table 3 may help in elaborating hypotheses on the evolution of these elements in insect genomes. The highest number of total ncRNAs in insect genomes was identified in the *D. melanogaster* genome, where it is close to 30% of the total number of protein coding genes. Evidently, this is due to the extensive genetics and genomics work done by the community that allowed the annotation of these loci. Nonetheless, what is surprising is the apparent considerable variation in the number of ncRNA genes seen among hymenopteran species. The fact that the numbers are most divergent for the lncRNAs is actually not surprising, as these cannot be annotated by customary similarity-based algorithms, but there is also considerable variation in the numbers of tRNA, snRNA, and miRNA loci in these genomes. Considering this, the variation denoted in Table 3 with respect to ncRNA gene numbers is, in fact, a glimpse into a major lacuna for hypotheses on insect genome evolution.

**Table 3:** Number of *F. varia* ncRNAs compared with the known ncRNAs from other insect genomes

RNA families	***F. varia***	***A. mellifera***	***B. impatiens***	***N. vitripennis***	***S. invicta***	***A. cephalotes***	***D. mel***	***A. gambiae***	***B. mori***
	v1.1	4.5	BIMP_2.0	2.1	Si_gnG	1.0	BDGP6	AgamP4	ASM15162v1
tRNA	180	193	216	215	390	290	314	463	427
snRNA	53	24	56	41	24	20	31	38	488
rRNA	21	56	93	47	65	32	147	78	110
miRNA	103	256	65	106	207	46	542	162	74
sno/scaRNA	9	5	9	8	7	9	288	12	8
lncRNA	1580	1	1	1			2776		
Total	1946	539	448	426	1379	449	4098	767	1122

### Genome organization, synteny, and repetitive genomic components

**Fig. 3:**
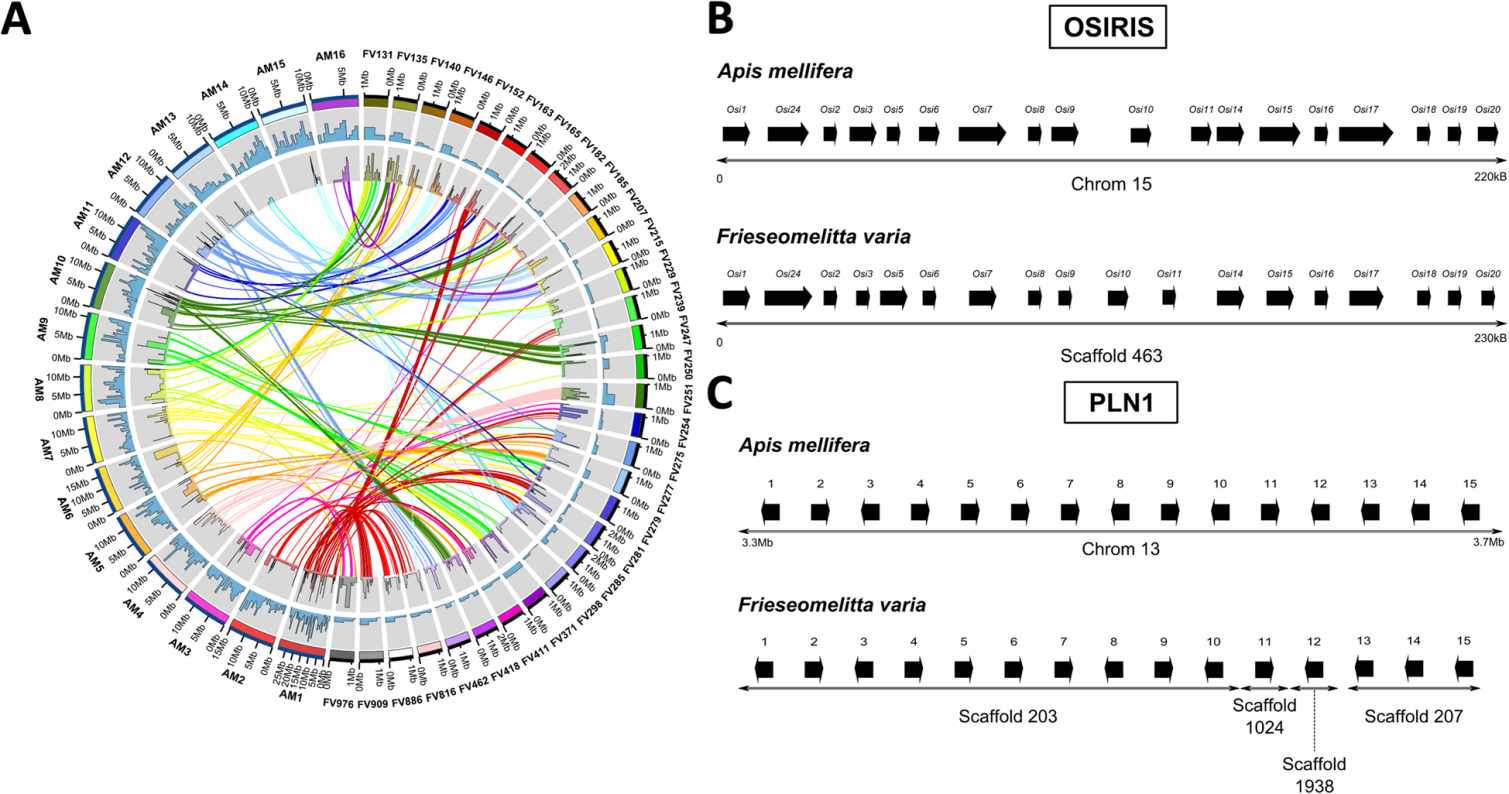
Genome synteny between *Apis mellifer*a and *Frieseomelitta varia*. **a** Mapping of *F. varia* genome scaffolds to *A. mellifera* linkage groups (chromosomes). **b** and **c** Gene clusters with conserved synteny in the *Frieseomelitta varia* genome. **b** Osiris gene cluster synteny with complete conservation in overall cluster size (230/220 kB), gene number and order, as well as gene orientation in the linkage groups of *F. varia* (scaffold 463) compared with *A. mellifera* (chromosome 15). **c** High degree of synteny in *A. mellifera* genes mapped in the *pln1* QTL for the pollen hoarding colony behavioral syndrome in comparison with the genomic localization of their orthologs in the *F. varia* genomic scaffolds 203 and 207

For an overview of the general genome structure we plotted the orthologous genes predicted in the scaffolds of *F. varia* against their respective position in the linkage groups of the honey bee genome. In the Circos plot (Fig. 3a), the same-colored lines connect orthologs of the two species with regard to their respective genomic localization based on linkage groups, which are chromosomes in the case of *A. mellifera* and scaffolds for *F. varia*. For example, most of the orthologs on the FV909, FV816, and FV163 scaffolds of *F. varia* mapped all to *A. mellifera* chromosome 1 (AM1). Similarly, most of orthologs located on FV418, FV247, and FV182 were found on AM10. With this in mind, we next conducted a more in-depth analysis into gene clusters that are known to play important roles in insect and, especially so, in bee biology: the Major Royal Jelly Protein (MRJP) family, the Osiris gene cluster, and the Pln1genes identified in a QTL of the pollen hoarding syndrome of honey bees.

The genes encoding MRJP/MRJP-like proteins of bees are inserted within the cluster of Yellow genes, specifically between *yellow e3* and *yellow h* [77]. But while the MRJP gene family has undergone a taxon-specific expansion in the genus *Apis*, consisting of a tandem array of nine functional MRJPs [77, 78], all the other corbiculate bees, as well most ants, with the exception of *Linepithema humile,* only have a single *mrjp-like* gene at this genomic location [8]. This genomic architecture was also found in the *F. varia* genome, with a single copy of an MRJP gene similar to *Apis mrjp-9* (*mrjp9-like*) being flanked by the two above-mentioned Yellow genes.

In the overall synteny analysis, the Osiris gene cluster stood out because of its high degree of structural genomic conservation. Figure 3b shows the organization for the *F. varia* Osiris gene cluster in comparison to *Apis mellifera*, not only in overall cluster size (230 vs. 220 kb), but also in terms of gene number and order, as well as transcriptional direction. Osiris genes are a highly conserved cluster of ~ 20 genes covering a genomic region of ~ 160 kb [79]. They are thought to have originated from gene duplications in early insects about 400 mya [4, 79] and to be related to insect wing evolution and radiation.

Compared to the conserved MRJP/MRJP-like and Osiris genes, a clearly unexpected result coming out of the synteny analysis was the finding for the genes located in the *pln1* QTL of the honey bee. This QTL was identified through a selection program over various generations for high vs. low levels of pollen collection and storage [80], which is a colony behavioral trait of honey bee workers. Such divergence in pollen hoarding behavior was subsequently found to have a strong association with gustatory responses and reproductive traits of *A. mellifera* workers [81–83]. In our genomic synteny analysis, the genes of the *pln1* QTL region on chromosome 15 of the honey bee showed a high degree of conservation in both gene number and order in the *F. varia* genomic scaffolds, especially so in scaffolds 203 and 207 (Fig. 3c). Interestingly, for the genes mapped to the other two pollen hoarding QTLs of the honey bee, *pln2* and *pln3* [80], we could not find such a strong linkage in the *F. varia* genome.

In terms of repetitive genomic components, we identified a total of 169,371 elements in the *F. varia* genome, belonging in majority to unknown elements. Specific transposable elements (LTR, LINE, SINE and DNA) represent 27.4% of the identified elements. A complete list of the identified repetitive genomic components, their respective numbers, cumulative length and % of the genome is presented in Table 4. In total, the repetitive genome represents 39.1 Mbp.

**Table 4:** Type and number of repetitive genomic elements

Order	Superfamily	Total	Cumulative length (Mb)	Cumulative length (bp)	% Genome
DNA	Unclassified	290	0.075844	75,844	0.02758
	CMC-Chapaev-3	63	0.007718	7718	0.00281
	Crypton	1	0.000084	84	0.00003
	Crypton-V	188	0.045662	45,662	0.01660
	hAT	114	0.017705	17,705	0.00644
	hAT-Ac	1896	0.706924	706,924	0.25706
	hAT-Blackjack	107	0.019388	19,388	0.00705
	hAT-Charlie	1533	0.422873	422,873	0.15377
	hAT-Tip100	99	0.021175	21,175	0.00770
	Kolobok-Hydra	2433	1.172149	1,172,149	0.42624
	Kolobok-T2	44	0.012426	12,426	0.00452
	Maverick	145	0.07668	76,680	0.02788
	Merlin	423	0.051174	51,174	0.01861
	MuLE-MuDR	57	0.011788	11,788	0.00429
	PIF-Harbinger	262	0.077291	77,291	0.02811
	PIF-Spy	127	0.017818	17,818	0.00648
	PiggyBac	3472	0.688623	688,623	0.25041
	Sola-1	143	0.036072	36,072	0.01312
	TcMar-Fot1	241	0.081897	81,897	0.02978
	TcMar-ISRm11	479	0.110088	110,088	0.04003
	TcMar-Mariner	3520	0.808834	808,834	0.29412
	TcMar-Pogo	43	0.00506	5060	0.00184
	TcMar-Stowaway	67	0.015985	15,985	0.00581
	TcMar-Tc1	21,131	5.625815	56,25,815	2.04575
	TcMar-Tc4	2991	0.61903	619,030	0.22510
	TcMar-Tigger	640	0.128285	128,285	0.04665
LINE	CR1	161	0.04403	44,030	0.01601
	I	2503	0.977458	977,458	0.35544
	I-Jockey	951	0.715998	715,998	0.26036
	L1	38	0.012373	12,373	0.00450
	R1	262	0.104838	104,838	0.03812
	R1-LOA	40	0.008446	8446	0.00307
	R2-NeSL	64	0.027806	27,806	0.01011
	RTE-X	522	0.20592	20,5920	0.07488
LTR	Unclassified	40	0.002817	2817	0.00102
	Copia	35	0.026156	26,156	0.00951
	ERV1	285	0.024605	24,605	0.00895
	ERVK	2	0.000117	117	0.00004
	ERVL	21	0.00166	1660	0.00060
	Gypsy	679	0.193441	193,441	0.07034
RC	Helitron	324	0.123596	123,596	0.04494
Retroposon	SVA	1	0.000144	144	0.00005
Satellite	Unclassified	720	0.371618	371,618	0.13513
	acro	127	0.009609	9609	0.00349
Simple_repeat	Unclassified	437	0.044859	44,859	0.01631
SINE	rRNA	129	0.031216	31,216	0.01135
	snRNA	149	0.021057	21,057	0.00766
srpRNA	Unclassified	1	0.000289	289	0.00011
tRNA	Unclassified	191	0.01394	13,940	0.00507
Unknown	Unclassified	121,180	25.279287	25,279,287	9.19247
**Total**		169,371	39.097668		14.21733

### The mitochondrial genome

We assembled the mitochondrial genome of *F. varia* from the whole genome sequencing reads and validated it with REAPR, which showed 57.53% of error-free bases. Also, no errors were identified, as indicated by the reported REAPR fragment coverage distribution (FCD) error, and there was no evidence of local misassemblies. In addition, we checked whether the sites showing rearrangements were consistent with the position of each pair of paired-end reads. This showed that at least 146 paired-end reads were found between sites, therefore confirming the sites with genes rearrangement.

**Fig. 4:**
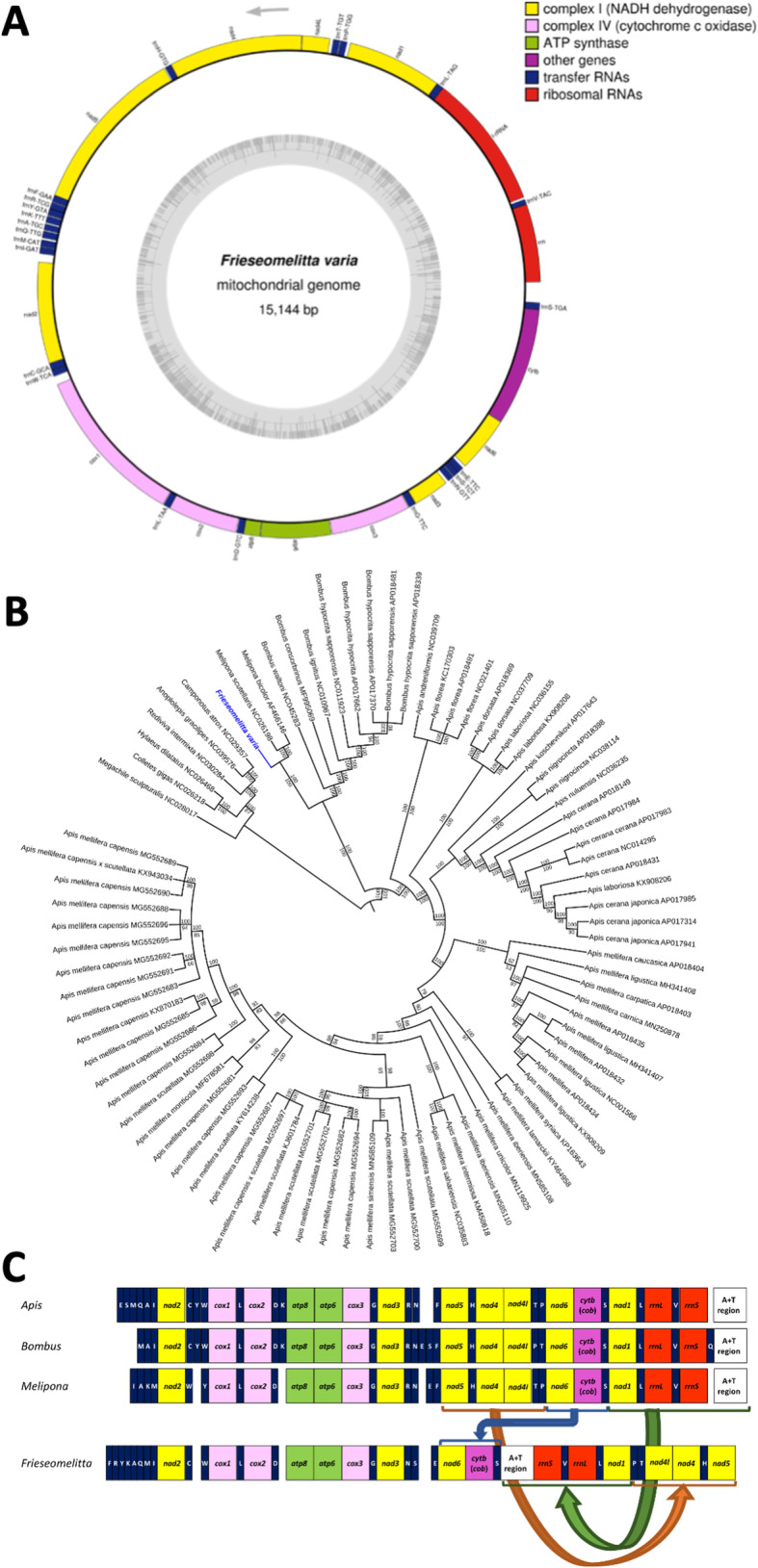
The mitochondrial genome of *Frieseomelitta varia.***a** Schematic representation of the circular mitochondrial genome. The arrow indicates transcription direction of the protein-coding genes. The genes encoding Complex I proteins are in yellow, those for Complex II are in pink, ATP synthase is in green, other protein coding genes are in purple, tRNAs are in blue, and rRNAs are in red. **b** Phylogenetic tree based on the complete mitochondrial genomes dataset of Apoidea and two ant species, showing proximate relation between the *F. varia* mitogenome with those of the two other stingless bees, *Melipona bicolor* and *M. scutellaris*. The tree was generated by Bayesian Inference; the values above the branches show the posterior probability for Bayesian Inference and values below the branches represent bootstrap support values of the Maximum Likelihood analyses. The position of *F. varia* is shown in blue. **c** Linear, schematic representation of the mitochondrial genome rearrangements inferred for the mitogenome of *F. varia* in comparison to the mitochondrial genomes of three other corbiculate bee genera, *Apis*, *Bombus* and *Melipona*. The colors of the genes correspond to the respective functional groups shown in the mitochondrial genome map. The blue arrow indicates the block of genes that showed rearrangement in gene order, and the green and orange arrows represent genes that underwent both rearrangement and inversion

As a result, the *F. varia* mitochondrial genome was found to consist of 15,144 bp and as having an A + T-rich nucleotide composition (87.8%), like in other insect species [84]. We identified all the 13 protein-coding genes, the 22 tRNA and 2 rRNA genes, as well as the non-coding control region. A map of the *F. varia* mitogenome is shown in Fig. 4a. All the 13 protein-coding genes (7 NADH dehydrogenases, 3 cytochrome C oxidases, 2 ATPases and cytochrome b) are encoded in the forward strand, and all have an initiation codon typical for invertebrates. The A + T-rich control region for the initiation of replication is located between the rRNA rrnS and the tRNA trnS-TGA.

The alignment of all mitochondrial genome genes (protein-coding genes, tRNAs and rRNAs) of 84 species resulted in a concatenated matrix of 16,499 bp, 12,008 bp, and 1749 bp length, respectively. The results for all trees using the two methods (ML and BI) were similar (Fig. 4b and Figures S2, S3, S4). Only for the tRNA trees there was less phylogenetic resolution, as indicated by tree topology and low branch support using both (RAxML and BI) methods (Figures S5 and S6).

The phylogenetic analysis indicated that the *F. varia* mitogenome is sister to those of the stingless bees *Melipona bicolor* and *Melipona scutellaris* (Fig. 4b), corroborating the results of a previous study based on mitochondrial 16S rDNA sequences [85].

In this context, a finding of particular interest was a major rearrangement in gene order in the *F. varia* mitogenome compared to *Apis, Bombus,* and even to the genus *Melipona* (Fig. 4c)*.* We found a drastic shuffling in gene positions for *nad1*, *nad4*–*6, cytb,* for the two rRNAs, as well as for the A + T-rich non-coding control region. Gene translocations have previously been described in other bee and wasp genomes [86, 87], and were also reported for *M. bicolor* [88].

### Gene families and functional groups of specific interest

*Transcription factors and epigenetic modifier genes*

By scanning the predicted protein sequences against the Pfam-A database using the hmmscan function, followed by the curation against the Transcription factor database v2.0 we identified 36 types of basal and 96 types of other transcription factor (TF) domains (Table 5). Among the basal TFs, the predominant ones were TFIIS_C, TAFH, TBP, and TF Zn Ribbon, which is a result similar to the one reported for the silk moth, *Bombyx mori* [40]. The predominant ones among the other types of TFs were zf-C2H2, Homeodomain, HLH, zf-C3HC4, and PHD, which is similar to the figure shown for the 10 previously sequenced bee species [8].

**Table 5:** Genes containing basal and other transcription factor (TF) domains identified in the *Frieseomelitta varia* genome

TF domain	Number of TF domain types	Number of genes with TF domains	Top TF domains	
Basal	38	50	*TFIIS_C*	4
			*TAFH*	3
			*TBP*	3
			*TF Zn Ribbon*	3
Other	96	589	*zf-C2H2*	161
			*Homeodomain*	76
			*HLH*	41
			*zf-C3HC4*	29
			*PHD*	28

Epigenetic mechanisms have emerged as important regulators of reproduction and behavior in social insects [89–91] Concerning genes involved in epigenetic modification, we identified a complete set of DNA methyltransferases (DNMTs) genes and genes associated with histone post-translational modifications (HPTM) in the *F. varia* genome. The sequences of these genes showed higher similiarity scores to their orthologs in *M. quadrifasciata* and the two bumble bee species for which a complete genome sequence is available [8, 68] than to those of *A. mellifera*.

#### *Genes related to reproduction*

The phenomenon of complete worker sterility in the genus *Frieseomelitta* made us take a closer look at genes and gene families related to reproductive processes in insects. From literature and GO searches we compiled a list of genes with known or predicted functions in the activation of the insect ovary. This list comprises a set of 127 genes in *F. varia,* and these are represented in very similar numbers in the genomes of other bees, as well as in the parasitic wasp *Nasonia vitripennis* and the fruit fly (Table 6). Among these, 61 are members of the core sets of major signaling pathways, such as Hippo, insulin/IGF, TGF-β, Wnt, and Notch. The gene models of these 61 core genes (in KEGG) were retrieved from the 10 annotated bee genomes [8], the *Euglossa dilemma* genome [69], the *A. cerana* genome [9] and the *A. dorsata* genome, and were compared with the respective *F. varia* gene models.

**Table 6:** Comparison of gene numbers with GO-BP terms associated with reproductive process in families of bees (Apidae, Megachilidae, Halictidae), the parasitic wasp *Nasonia vitripennis* and *Drosophila melanogaster*

Species	Genes
Apidae	
*Apis mellifera*	149
*Apis dorsata*	132
*Apis florea*	136
*Bombus impatiens*	140
*Bombus terrestris*	138
*Eufriesea mexicana*	117
*Euglossa dilemma*	91
***Frieseomelitta varia***	**127**
*Melipona quadrifasciata*	124
*Habropoda laboriosa*	129
Megachilidae	
*Megachile rotundata*	123
Halictidae	
*Dufourea novaeangliae*	126
*Lasioglossum albipes*	127
Pteromalidae	
*Nasonia vitripennis*	130
Drosophilidae	
*Drosophila melanogaster*	106

**Fig. 5:**
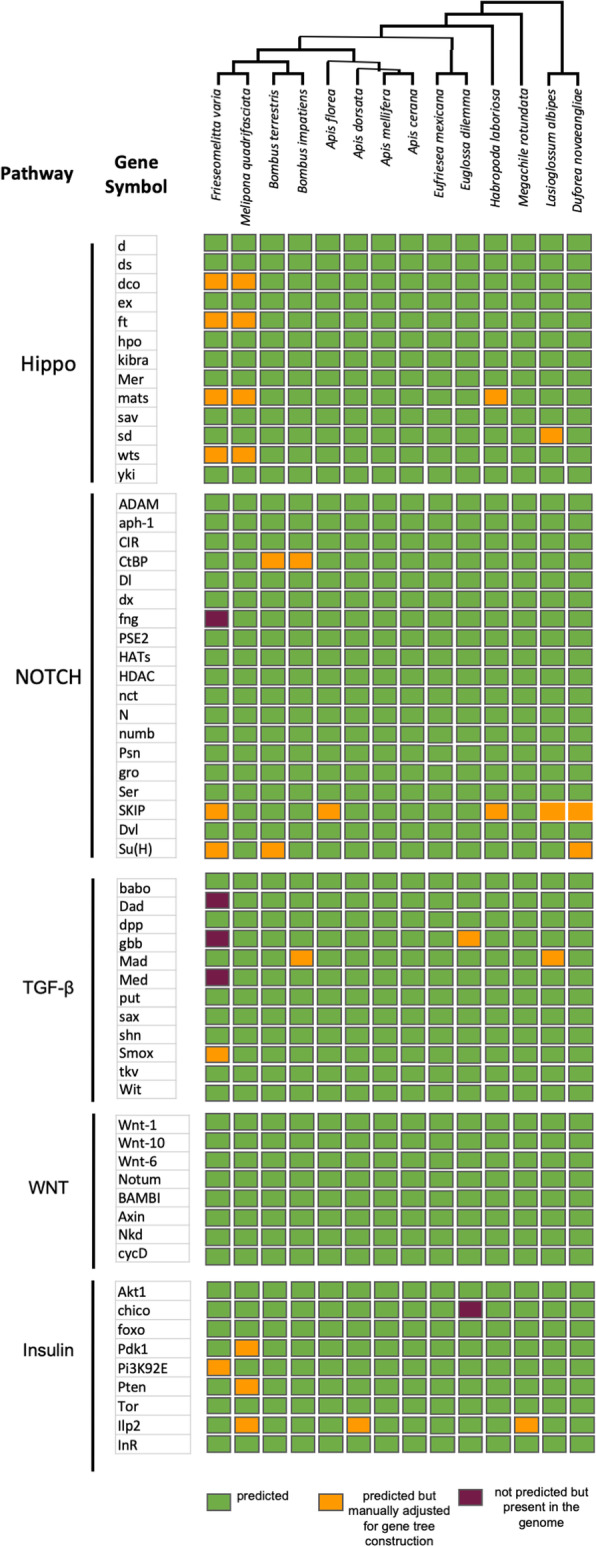
Overwiew on signaling pathways core genes in bee genomes. Shown are the core genes of the Hippo, Notch, TGF-β, Wnt, and insulin signaling pathways in the genome of *F. varia* compared to 13 other sequenced and annotated bee genomes. **Hippo**: d: *dachs*, ds: *dachsous*, dco: *discs overgrown*, ex: *expanded*, ft.: *fat*, hpo: *hippo*, kibra: *Kibr*, mats: *mob as tumor suppressor*, Mer: *Merlin*, sav: *Salvador*, sd: *Scalloped*, wts: *Warts*, yki: *Yorkie.***Notch: **ADAM: *ADAM 17-like protease*, aph-1: *anterior pharynx defective 1*, CIR: *corepressor interacting with RBPJ 1*, CtBP: *C-terminal binding protein*, Dl: *delta*, dx: *deltex*, fng: *fringe*, PSE2: *presenilin enhancer*, HATs: *histone acetyltransferase KAT2A*, HDAC1: *histone deacetylase 1*, nct: *nicastriin*, N: *notch*, numb: *numb*, Psn: *presenilin*, gro: *groucho*, Ser: *serrate*, SKIP: *puff-specific protein Bx42*, Dvl: *segment polarity protein dishevelled homolog DVL-3*, Su(H): *suppressor of hairless*. **TGF-β:** babo: *baboon*, Dad: *Daughters against dpp*, dpp: *decapentaplegic*, gbb: *glass bottom boat*, Mad: *Mothers against dpp*, Med: M*edea*, put: *punt*, sax: *saxophone*, shn: *schnurri*, Smox: *Smad on X*, tkv: *thickveins*, wit: *wishful thinking*. **WNT:** Wnt1: *wingless-type MMTV integration site family*, *member 1*, Wnt10: *Wnt oncogene analog 10*, Wnt6: *Wnt oncogene analog 6*, Notum: *Notum*, Bambi: *Bambi*, Axn: *Axin*, nkd: *naked cuticle*, CycD: *Cyclin D*. **Insulin**: akt1: *Akt1*, chico: *chico*, foxo: *forkhead box*, *sub-group O*, Pdk1: *Phosphoinositide-dependent kinase 1*, Pi3K92E: *Pi3K92E*, Pten: *Phosphatase and tensin homolog*, Tor: *Target of rapamycin*, Ilp2: *Insulin-like peptide 2*, InR: *Insulin-like receptor*

**Fig. 6:**
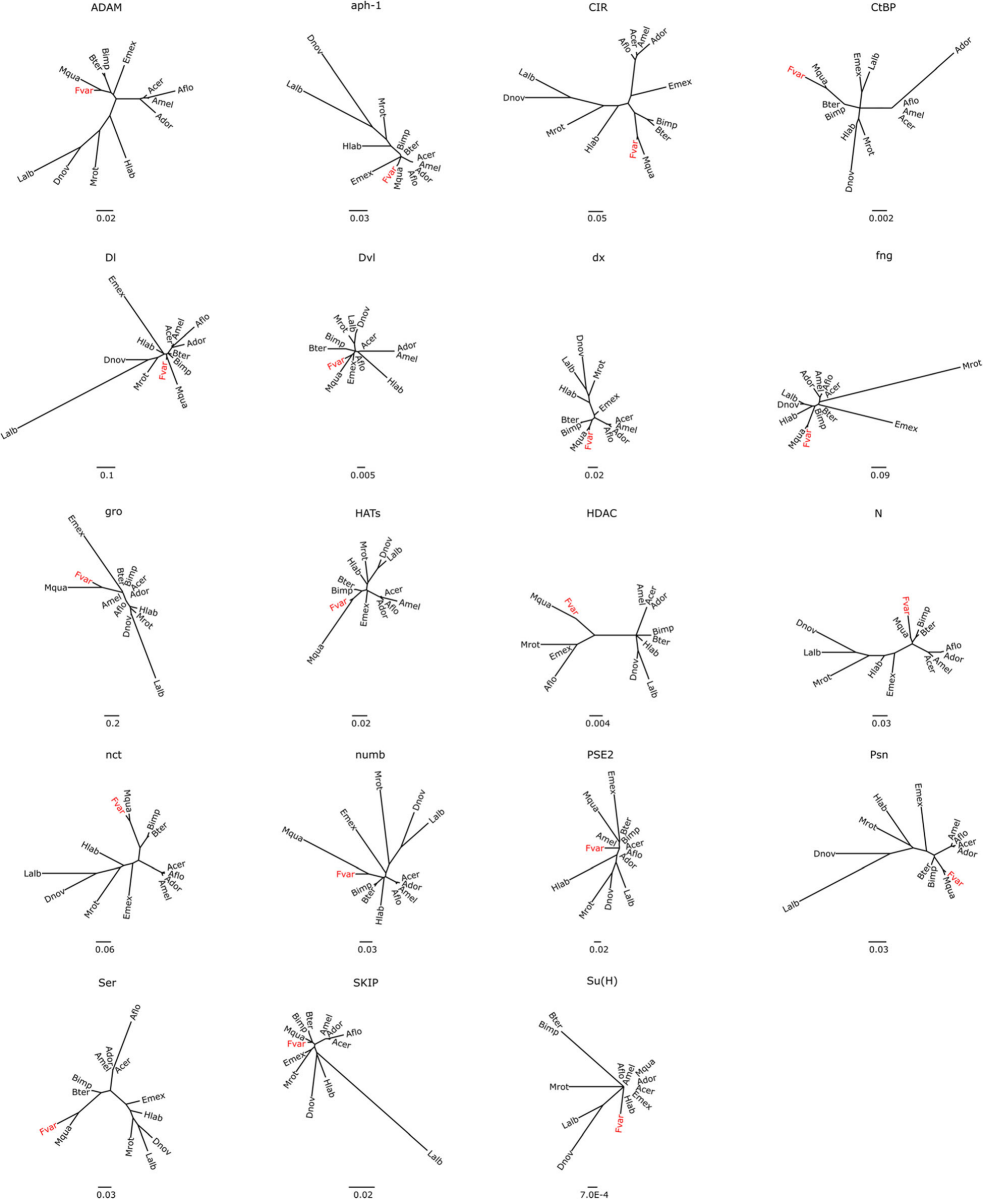
Gene tree for the key gene set of Notch signaling in bees. Orthologous aminoacid sequences were aligned using MAFFT, and the tree was generated using a Maximum Likelihood approach (1000 replicates). In red are the respective orthologs for *F. varia*. Gene names are abbreviated as: ADAM, *ADAM 17-like protease*; aph-1, *anterior pharynx defective 1*; CIR, *corepressor interacting with RBPJ 1*; CtBP, *C-terminal binding protein*; Dl, *delta*; Dvl, *segment polarity protein dishevelled homolog DVL-3*; dx, *deltex*; fng, *fringe*; gro, *groucho*; HATs, *histone acetyltransferase KAT2A*; HDAC1, *histone deacetylase 1*; N, *notch*; nct, *nicastriin*; numb, *numb*; PSE2, *presenilin enhancer*; Psn, *presenilin*; Ser, *serrate*; SKIP, *puff-specific protein Bx42*; Su(H), *suppressor of hairless*. Species names are given as three letter acronyms: Acer, *Apis cerana*; Ador, *Apis dorsata*; Aflo, *Apis florea*; Amel, *Apis mellifera*; Bimp, *Bombus impatiens*; Bter, *Bombus terrestris*; Dnov, *Dufourea novaeangliae*; Edil, *Euglossa dilemma*; Emex, *Eufriesea mexicana*; Fvar, *Frieseomelitta varia*; Hlab, *Habropoda laboriosa;* Lalb, *Lasioglossum albipes*; Mrot, *Megachile rotundata*; Mqua, *Melipona quadrifasciata.* Scale bars indicate the number of substitutions per site

A summary report representing the phylogenetic gene tree reconstructions for all these pathways is shown in Fig. 5 and details are shown for the key gene set of the Notch pathway (Fig. 6). All the respective core genes were identified in the *F. varia* genome, and the automatically predicted gene models turned out to be correct for 50 (80%) of these, while 8 genes (13%) required corrections in the automatically predicted gene models, and 4 (7%) that had not been predicted were found by manual blastp searches. As it turned out, their sequences were fragmented and represented on different scaffolds. Our phylogenetic tree reconstruction for the Notch pathway genes in bees (Fig. 6) confirmed that the *F. varia* orthologs were generally closely related to those of *M. quadrifasciata*.

#### *Immunity-related genes*

**Fig. 7:**
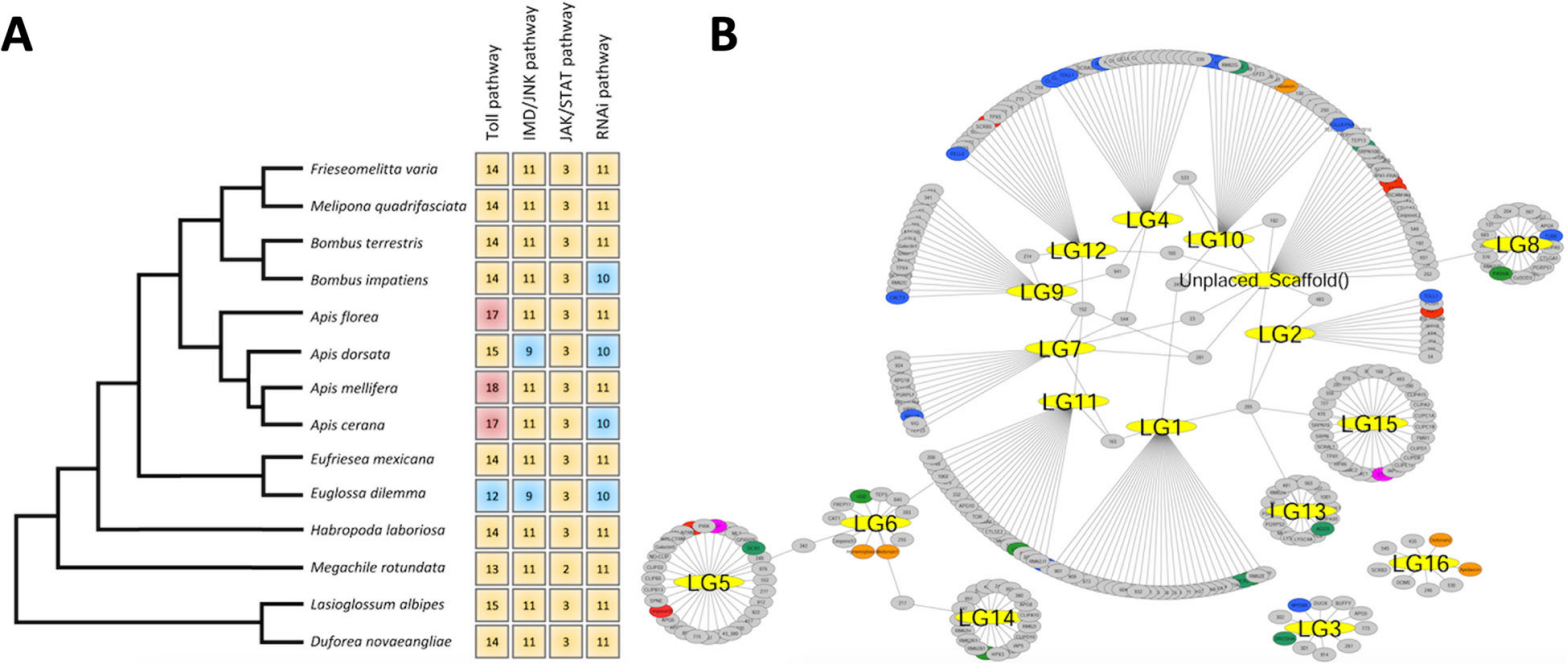
Immunity related genes in *Frieseomelitta varia*. **a** Representation of gene numbers associated with the canonical innate immunity pathways and RNAi in sequenced bee genomes. A red background means more genes and a blue one less genes for the respective pathway in a given species. **b** Interaction network relating the *F. varia* immunity genes and their respective genomic scaffolds and their localization with their *A. mellifera* orthologs and respetive chromosome (LG1 – LG16) localization. Shown are only *F. varia* scaffolds with more than one predicted immunity gene. Blue: Toll pathway; red: IMD pathway; pink: JAK/STAT pathway; orange: AMPs; green RNAi genes

We screened the *F. varia* genome for the presence of immunity related genes. Of the 190 immune response genes predicted for *A. mellifera* [8], we found 174 homologs in the *F. varia* genome (Table 7, Figures 7a, Figue S7 and S8). Worthy of note is that we did not find orthologs of the genes encoding Cactus-2 and Cactus-3, Dorsal-2 (Relish1B), as well as of some apoptosis inhibitors (IAPs), Toll8 and CLIP. This could be due to a lack of coverage in DNA sequencing, but there was also no evidence for the respective transcript in the RNA libraries used in the mapping procedure. Furthermore, some of these genes also did not have identifiable orthologs in certain other bee genomes. An interaction network for the immunity genes of *F. varia* showing their scaffold localization in relationship to the 16 honey bee chromosomes is shown in Fig. 7b.

**Table 7:** Number of genes related to immunity pathways and functions in 11 bee species with complete genome information, including 10 previously published species [8, 67] and *Frieseomelitta varia* (in bold)

Species	Toll pathway	IMD pathway	Jak/STAT	Recognition proteins	RNAi machinery	Signaling proteins	Effector proteins	Total number of genes
Apidae								
*Apis florea*	17	11	3	21	11	36	10	178
*Apis mellifera*	18	11	3	21	11	38	13	190
*Bombus impatiens*	14	11	3	20	10	31	9	171
*Bombus terrestris*	14	11	3	20	11	33	8	172
*Eufriesea mexicana*	14	11	3	18	11	32	10	168
***Frieseomelitta varia***	**14**	**11**	**3**	**20**	**11**	**32**	**10**	**174**
*Melipona quadrifasciata*	14	11	3	20	11	33	10	172
*Habropoda laboriosa*	14	11	3	21	11	33	9	174
Megachilidae								
*Megachile rotundata*	13	11	2	19	11	31	9	167
Halictidae								
*Dufourea novaeangliae*	14	11	3	19	11	32	7	167
*Lasioglossum albipes*	15	11	3	19	11	34	9	172

#### *Neurogenesis-related genes*

The exceptional performance of honey bee workers in learning and memory task assays has been a driver for high-throughput sequencingefforts and the establishment of a microarray platform since the early days of honey bee genomics [92, 93]. There are two central questions, one is how the behavioral sequencing efforts and the establishment of a microarray platform since the early days of honey bee genomics [92, 93]. There are two central questions, one is how the behavioral plasticity and task switching in adult workers is reflected in transcriptional brain profiles [94, 95], and the other is how gene expression in the larval brain may underlie the development of caste- and sex-specific brain anatomies [96, 97].

**Fig. 8:**
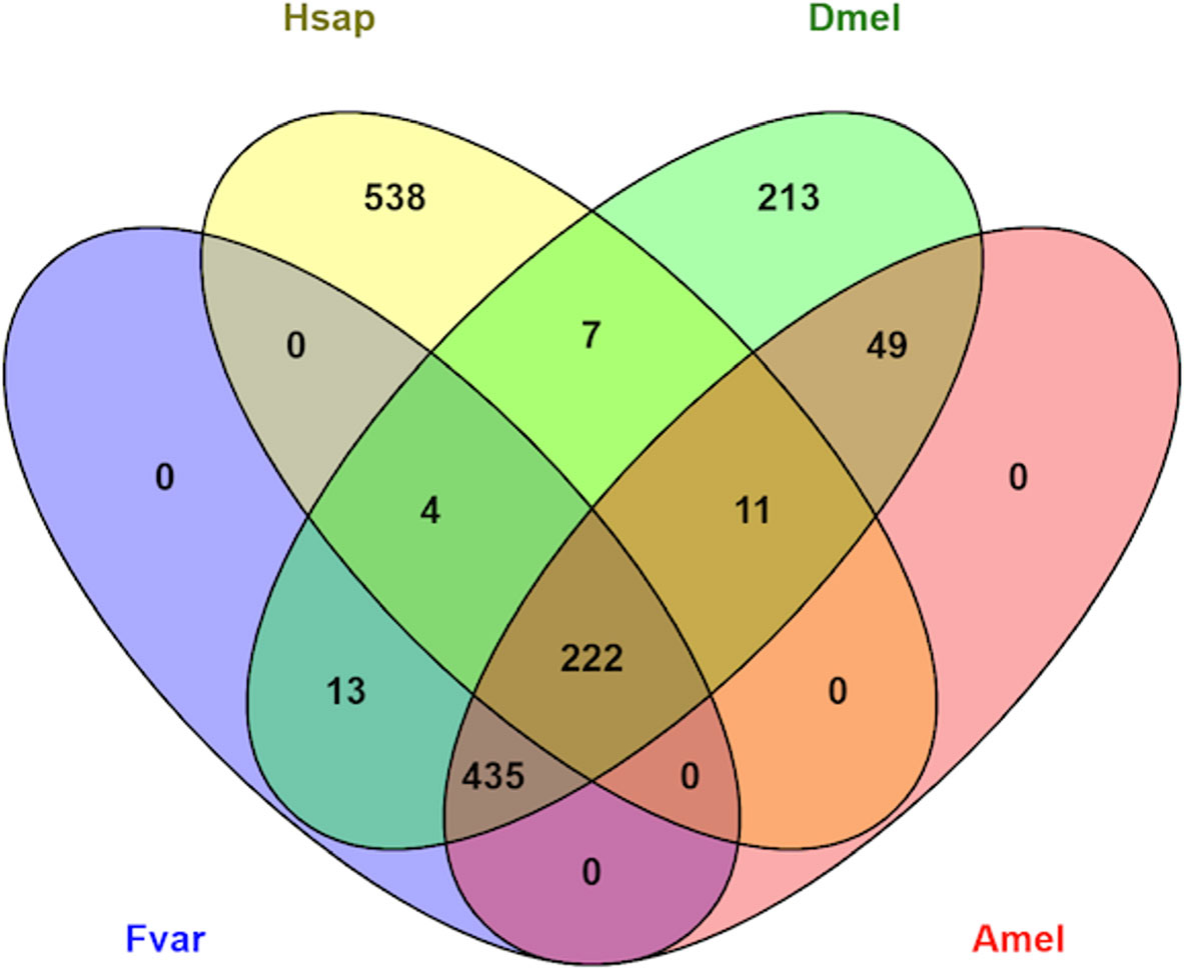
Neurogenesis-related genes. Venn diagram of direct orthologs of neurogenesis-related genes in the human (Hsap), *Drosophila* (Dmel), honey bee (Amel), and *F. varia* (Fvar) genomes

As genes involved in neurogenesis are candidates for the wiring and rewiring of neuronal networks, our aim here was to get an overview on neurogenesis-related genes in bees and, especially so, in the *F. varia* genome. Using the Gene Ontology Biological Process term neurogenesis (GO: 0022008) for the identification of human and *Drosophila* orthologs in 11 bee genomes, we retrieved a total of 1586 genes in the human genome and 954 genes in the fruit fly, representing 7.94 and 5.36%, respectively in terms of total gene numbers (Table 8). In the sequenced bee genomes, the number of neurogenesis-associated genes varies between 672 and 732, with respective percentages between 5.31 and 5.61. The relatively high percentage of these genes in the *F. varia* genome is intriguing, but, at present, it should not be over-interpreted, as this may change once future annotation efforts come up with a refined total gene number estimate. The Venn diagram comparing sequence similarity of neurogenesis-related genes annotated in the *F. varia* genome with those in the human genome, the fruit fly, and the honey bee (Fig. 8) shows that most of the *F. varia* neurogenesis genes have close orthologs in the honey bee (64.5%) or with the honey bee and the fruit fly combined (32.9%).

**Table 8:** Neurogenesis-related genes and percentage relative to total gene number in bee genomes compared to humans and *Drosophila*

Species	Neurogenesis-related genes	Total gene number	neurogenic/total genes (%)
*Homo sapiens*	1586	19,986	7.94
***Frieseomelitta varia***	**674**	**10,346**	**6.51**
*Habropoda laboriosa*	715	12,279	5.82
*Dufourea novaeangliae*	721	12,453	5.79
*Eufriesea mexixana*	696	12,022	5.79
*Bombus impatiens*	732	13,050	5.61
*Megachile rotundata*	717	12,770	5.61
*Lasioglossum albipes*	722	13,448	5.37
*Drosophila melanogaster*	954	17,792	5.36
*Bombus terrestris*	672	12,648	5.31
*Apis mellifera*	717	15,314	4.68
*Melipona quadrifasciata*	703	15,368	4.57
*Euglossa dilemma*	679	15,904	4.27

#### *Chemosensory repertoire and circadian rhythm genes*

To explore the chemosensory repertoire in bees, we used information from two phylogenetically close bee species, *A. mellifera* and *M. quadrifasciata* [98, 99], as input to identify the gene family sizes of odorant receptors (ORs), odorant binding proteins (OBPs), chemosensory proteins (CSPs), gustatory receptors (GRs) and ionotropic receptors (IRs) in the *F. varia* genome. In addition, we searched for protein-coding sequences of SNMPs [Sensory Neuron Membrane Proteins, Group 3 (SNMP1 and SNMP2)] in the genomes of these three bee species, and we also updated information on IR gene numbers for *A. mellifera* and *M. quadrifasciata*.

We performed a thorough manual curation that included multiple and local alignments and reciprocal BLAST searches in order to: (i) check the existence of non-automatically predicted gene models, (ii) obtain information on protein sequence lengths and structure of the coding regions, (iii) correct gene models when needed, and (iv) screen for the presence of the following canonical domains: 7tm_6 (pfam02949) for ORs, PBP_GOBP (pfam01395) for OBPs, OS-D (pfam03392) for CSPs, 7tm_7 (pfam08395) for GRs, PBP2_iGluR (cd13717) for IRs, and CD36 (pfam01130) for SNMPs 1 and 2.

The gene numbers for each chemosensory family that we identified in the *F. varia* genome (Table 9) are based on a more conservative approach than that of Brand and Ramírez [99], which for ORs used the earlier prediction made by Robertson and Wanner [98]. Specifically, we excluded: pseudogenes, protein-coding sequences without canonical domains, partially predicted protein sequences (incomplete ORFs or lacking an initial methionine residue), and/or sequences that presented poor alignments with those of the other bees. Notwithstanding, it is already possible to draw some conclusions. In accordance with previous studies [100, 101], we observed a variable degree of evolutionary conservation. For instance, sequence similarity for OBP and OR genes ranged from 54 to 73%, and 33 to 86%, respectively, except for the ORCO gene (also called OR2) that showed 95% similarity.

**Table 9:** Comparison of the number of genes encoding for odorant receptors (ORs), odorant binding proteins (OBPs), chemosensory protein (CSPs), gustatory receptors (GRs), ionotropic receptors (IRs), and sensory neuron membrane proteins (SNMPs) identified in the genomes of the honeybees (*Apis mellifera*) and two stingless bees (*Melipona quadrifasciata* and *Frieseomelitta varia*)

Family	*A. mellifera*	*M. quadrifasciata*	*F. varia*	References
ORs	177	196	–	[98, 99]
	–	–	102	This study
OBPs	21	14	–	[99]
	–	–	8	This study
CSPs	6	6	–	[99]
	–	–	5	This study
GRs	14	16	–	[99]
	–	–	10	This study
IRs	10	10	–	[99]
	16	17	15	This study
SNMPs, Group 3	–	–	–	[99]
	2	3	3	This study

With respect to circadian rhythm genes, we manually curated the automatially predicted gene models of eight canonical clock genes in the genome of *F. varia* based on orthology to *A. mellifera* and *D. melanogaster*: *clock, cycle, period, cryptochrome mammalian-like, par domain protein, vrille, timeout2, and clockwork orange*. The gene models were all correct and consistent with those of other bee species.

## Discussion

### Genome assembly and genome organization

We report here the genome sequencing, assembly, and annotation results for the highly eusocial stingless bee *F. varia*, which, after *M. quadrifasciata*, is now the second stingless bee species with complete genomic information. For the nuclear genome we predicted an assembled genome size of 275 Mbp (and a GC content of 37% (Table 1). This genome size estimate fits in very well with previously reported estimates for solitary and social bees [2, 8, 68, 69, 102]. Nonetheless, it differs drastically from the size estimate of 450 Mb obtained by flow cytometry [103]. Since repetitive regions represent a challenge to genome assemblies from short Illumina reads, we used a k-mer counting approach to obtain a further genome size estimate. This resulted in a haploid genome length of 345.8 Mbp. Furthermore, our analysis of repetitive genomic components indicated a repeat genome size of 39 Mbp. So, at least part of the discrepancy between the flow cytometry estimate and the assembled genome size may be due to the high number of repetitive genomic components (Table 4). The k-mer-based genome size estimate minus repeats almost represents the assembled portion of the genome and, in line with BUSCO, the assembly, therefore, appears to be rather complete. Under- or overestimates of genome size based on flow cytometry results have previously been reported for *Bombus terrestris* [104].

The number of predicted protein-coding genes (10,526) is close to the original prediction for *A. mellifera* gene number [1], but ca. 30% lower than the subsequently revised gene number prediction [2], and also lower than the gene numbers predicted for the other sequenced bee genomes [8]. Nonetheless, it is close to the gene number predicted for the more recently sequenced subsocial carpenter bee *Ceratina calcarata* [102], which is considered a putative model species for understanding social evolution based on matrifilial association [105].

As the assembly for the *F. varia* genome, generated in two steps from Illumina reads (Table 1), is still rather fragmented, we conducted a synteny analysis as guidance for a future hybrid assembly, as recently completed for the honey bee [3]. This analysis showed that several genes placed on the smaller *F. varia* scaffolds had their respective orthologs placed together on one of the much larger, chromosome-size linkage groups of the honey bee (Fig. 3). Such correspondence in scaffold/chromosome localization for orthologous genes indicates a considerable degree of conservation in overall genome organization across bee genomes, despite the divergence time between the tribes Apini and Meliponini of more than 80 million years [5], and the differing ancestral haploid chromosome numbers between Apini (16) and Meliponini (range 8–18, with *n* = 9, *n* = 15, and *n* = 17 as the main groups, with an n = 15 for *F. varia* [106]). High conservation of the genetic structure was also observed between the genomes of *A. mellifera* and *B. terrestris* [103] and between *A. mellifera* and *E. dilemma* [69], suggesting slow genome evolution in Apidae. Furthermore, as a byproduct of this synteny analysis we were able to take a closer look at some gene clusters that are known play a role in insect and specifically bee biology, such as the Major Royal Jelly Proteins (MRJPs), the Osiris gene cluster, and the *pln1* QTL genes.

Concerning MRJP genes, already previous analyses [8, 77, 78] had shown that the tandem array of nine functional MRJP genes in the genus *Apis* represents a taxon-specific expansion expansion, and that all the other corbiculate bees, including stingless bees, have only one ortholog named *mrjp-like.* In honey bees, MRJPs are the main proteinaceous components in royal and worker jelly, but in larval food of the stingless bee *M. quadrifasciata* no MRJP-like proteins were detected by a proteomics analysis (S. Albert, G.J. Tibério, K. Hartfelder, unpublished data; MALDI/TOF/TOF analysis of trypsinated peptide fragments from SDS-PAGE gel pieces of the size range 20–60 kDa). Furthermore, we also could not identify an ortholog of the *apisimin* gene in the genomes of *F. varia* and *M. quadrifasciata.* Hence, the peculiar molecular architecture combining native MRJP1, apisimin and 24-methylene cholesterol in honey bee royal jelly [107] seems to be specific to the genus *Apis.* It is likely crucial for the viscosity of royal jelly that makes it possible that honey bees can rear their queen larvae in downward pointing royal cells [108]. In contrast, stingless bees rear their brood in horizontal cells, with the larvae floating on top of a defined portion of larval food. Their larval food also has a species-specific composition of proteins, but this is quite different from that of the honey bees [109]. With this in mind, the function of the *mrjp-like* gene in stingless bees deserves a closer look, especially considering the diverse modes of caste determination in the Meliponini.

The Osiris genes of *F. varia* (Fig. 3b) form a highly conserved cluster of 18 genes. An Osiris cluster is thought to have originated already very early in the evolution of winged insects [4, 79]. Astonishingly, despite this ancient conservation in genomic organization, the Osiris genes have no Gene Ontology terms associated concerning Molecular Function, and only a putative functional protein domain is predicted. Gene expression analyses, however, showed a correlation between neighborhood distances of the Osiris genes within the cluster to the timing of developmental events, especially so cuticle formation in the embryo and during metamorphosis, as well as differences in Osiris gene expression associated with caste development in social insects [110]. Besides their strong correlation with the timing of cuticle formation, co-expression analyses indicate that they are also involved in immune system functions and detoxification reactions in the red flower beetle, *Tribolium castaneum* [111].

As said, the finding of a conserved cluster of *pln1* QTL genes (Fig. 3c) was unexpected, as this QTL was identified through a selection program for pollen hoarding behavior in honey bees [80]. This is a colony behavioral trait hat was subsequently found to have a strong association with gustatory responses and reproductive traits of *A. mellifera* workers [81–83]. At present, without more in-depth analyses concerning the extent to which the *pln1* cluster may be conserved across bees, we can only speculate on the possible meaning of this architectural genomic conservation. The collection of pollen, which underlies the pollen hoarding syndrome in *A. mellifera*, was a, if not the crucial step in the life style transition that originated the bees from a wasp ancestor. A recent phylogeny proposed the tiny Ammoplanina wasps as the sister group of the bees (Anthophila) [112]. These wasps hunt thrips on flowers, a behavior which would have facilitated the transition from a predatory to a pollen collecting life style. This evolutionary scenario of the bees could explain the conservation seen in the *pln1* gene cluster, a genetic module that likely shifted and directed a wasp’s predatory food collecting behavior to a flower resource. Testing this hypothesis will, however, require further evidence from genomic comparisons across different families of bees and wasps.

### The mitochondrial genome

With the COX2 sequence of *Bombus hypocrita sapporensis* as seed, the mitochondrial genome of *F. varia* was extracted from the trimmed genomic paired-end reads and assembled into a circularized gene order using the organelle-specific software NOVOPlasty (Fig. 4a). The size of the *F. varia* mitochondrial genome, its A + T composition, and also the number of genes was found to be consistent with the mitochondrial genomes of other insects [84]. Furthermore, the alignment of the mitochondrial genes of bees of the family Apidae and their representation in a phylogenetic tree using Bayesian inference and Maximim Likelihood approaches (Fig. 4b and Figure S2-S6) showed high support for a sister group relationship of *F. varia* with the two stingless bees of the genus *Melipona*, for which mitochondrial genome assemblies are available [88, 113].

A remarkable finding was that of a major rearrangement in gene order in the *F. varia* mitogenome compared to members of three other tribes within the Corbiculata clade, *Apis, Bombus,* and *Melipona* (Fig. 4c)*.* Nonetheless, while tempting, it is still early to speculate whether stingless bees may be a hotspot in mitochondrial genome evolution. In the genus *Apis*, dozens of mitogenomes have been sequenced for practically all the species, subspecies, and even local populations, but with respect to stingless bee, we are aware of only three fully sequenced mitogenomes, including those of *M. bicolor* [88], *M. scutellaris* [113], and now *F. varia.* The finding of such extensive reshuffling in gene order should, thus, clearly be an incentive for future stingless bee mitogenome sequencing projects.

### Gene families and functional groups of specific interest

In the first comparative genomics study on social evolution in bees [8], transcription regulating factors, including transctiption factors (TFs) and their respective binding sites in *cis*-regulatory regions of potential target genes, as well as egigenetic modification and genes involved in these processes were pointed out as possible hallmarks. Hence, we performed here specific searches for TFs and epigenetic modifier genes in the *F. varia* genome. With regard to TF families we found figures very similar to the previously reported ones [8], and this was also the case for epigenetic modifier genes.

Amongst epigenetic mechanisms, DNA methylation is clearly the best studied in the context of reproduction and behavior in social insects [89]. Nonethelesse, one aspect that has recently come to light in a study on the emerging insect model organism *Tribolium castaneum,* namely the interaction between genes promoting histone post-translational modifications and juvenile hormone (JH) functions [114], could be specifically relevant with relation to reproduction and aging in highly social insects. JH is a key regulator of insect development and reproduction, as well as of caste development in social insects [115], and JH levels in the life cycle of a stingless bee, *M. scutellaris,* were recently determined [116]. Hence, we consider that epigenetic modifier genes interacting with key insect hormones and long non-coding RNAs [117] may also play a critical role in caste development and reproduction, especially so the phenomenon of complete worker sterility in *F. varia*.

For a better understanding of other key processes associated with hymenopteran eusociality, such as immune system functions, reproductive biology, nervous system organization, circadian rhythm and the chemosensory system, we performed specific searches for such genes in the *F. varia* genome. We conducted a manual curation of their automatically generated predictions and a phylogenetic comparison with their orthologs in the currently sequenced bee genomes.

As for immunity-related genes, we could clearly identify 174 of the 190 genes predicted in the honey bee genome, while for the remainder we could not find orthologs in the *F. varia* genome. This could be due to the fact that the *F. varia* genome is still split into many scaffolds, as well as to the fact that some of these “missing genes” were also not found in the genomes of other social bees. Nonetheless, the phylogenetic analyses for the primary immune gene families showed that the *F. varia* genes had their closest orthologs in *M. quadrifasciata* and the two *Bombus* species (Figures S7 and S8).

Considering the phenomenon of worker sterility in *F. varia* [23], based on literature searches and GO terms we compiled a list of 127 candidate genes that are involved in reproductive processes, especially ovary function in insects. Among these, approximately 50% are related to major signaling pathways. As shown in Fig. 5, not all the genes related to these signaling pathways had been correctly predicted, including *fringe*, which is an important member of the Notch pathway, and this would have been astounding, since the Notch pathway has recently been identified as a key regulator for differential ovarian activity in honey bee queens and workers [118]. Nonetheless, by a specific search we found this gene as split between two scaffolds. Hence, besides finding “missing genes” this strategy, though laborious, can help to bridge scaffolds.

Another aspect of prime importance in social insects is communication, and in this respect, bees, like most other insects, make extensive use of olfactory information in fundamental behaviors, such as food source location and recognition of conspecifics [119]. Social organization imposes an extra layer of complexity, including food source communication and brood care by workers, reproductive division of labor among queens and workers, nest defense, and in the adjustment in worker behavioral maturation with colony demography. Our results for *F. varia* are consistent with those reported for chemosensory genes in corbiculate bees and wasps, both in terms of gene numbers and the *in tandem* clustered organization in some of these [99, 119]. We also found a slightly larger number of SNMP genes in the two stingless bees (two SNMP1 and one SNMP2) compared to *A. mellifera* (one SNMP1 and one SNMP2), suggesting a possible gene loss in honey bees.

Integration of social behaviors among colony members also requires synchronization of circadian cycles. As such, the circadian clock of bees is thought to control most of the behavioral aspects of division of labor and synchronization of worker bee activities [120]. For *F. varia,* the molecular functioning and integration of clock genes in generating circadian rhythms is probably similar to *A. mellifera* [121], with a first autoregulatory feedback loop composed by Clock, Cycle, Period, and Cryptochrome mammalian-like, and a second autoregulatory feedback loop involving Clock, Par domain protein 1, and Vrille. Timeout 2, but not its paralog Timeless, is only present in Hymenoptera [122], and the *Drosophila* ortholog Clockwork orange appear to be involved in different clock functions [123, 124]. Chronobiology studies making use of this genomic information may provide important insights into the evolution of the different life styles seen in stingless bee species.

## Conclusions

The species *F. varia* was chosen for this genome project because of its peculiar reproductive biology. Different from most other stingless bee species, *F. varia* workers are completely sterile and, thus represent an extreme end in the queen/worker and worker/worker conflict of interest over male production in a bee colony. For insights into genomic signatures related to reproduction and social behaviors, we checked and manually curated hundreds of gene models predicted for genes related to reproductive functions, including several core signaling pathways, as well as genes related to immunity, neurogenesis and chemosensory processes. Furthermore, we generated predictions for genes containing transcription factor domains, repetitive genomic components, and non-coding RNAs, including over 1500 lncRNAs.

Specific highlights of the genome analysis were the finding of a complete and highly conserved Osiris gene cluster in a single scaffold of the *F. varia* genome and the conservation of the *pln1* QTL. The Osiris gene cluster is a unique characteristic of insect genomes. Yet, despite its ancient origin and high genomic conservation, the molecular functions of these genes are still enigmatic. Clearly unexpected was the conservation in the *F. varia* genome of the *pln1* QTL found in the honey bee. This QTL has been identified and described as a key element in the reproductive ground plan underlying the social behavior of honey bees. Clearly there was no *a priori *reason to believe that this gene cluster would be so conserved also in stingless bees, considering the divergence time of over 80 mya between honey bees and stingless bees and the much more recent origin of the genus *Apis* compared to the stingless bees. This suggests that a QTL with genes related to pollen hoarding behavior may have arisen already at an early time in the phylogeny of the Apidae and since remained stabilized.

In contrast to the examples of conservation in the nuclear genome, the mitochondrial genome of *F. varia* revealed major changes in gene order in comparison to the honey bee and also to the other two stingless bee mitogenomes sequenced so far. For *F. varia* we found evidence for major reshuffling events in gene order that should be of interest for the analysis of other Meliponini mitogenomes, as this group may represent a hotspot in mitogenome structural evolution.

With several hundred species distributed in 61 genera worldwide, the stingless bees are the only bees that equal honey bees in terms of social organization and complexity. Hence, their ecological and economic importance, as well as their biological variability should more than justify further genome sequencing efforts in the tribe Meliponini to reveal genomic signatures associated with the evolution of advanced sociality in this group, in comparison and contrast to the tribe Apini.

## Supplementary information

**Additional file 1 : Figure S1:** Multiple alignment (A) and Maximum Parsimony phylogenetic tree (B) of miR-34.

**Additional file 2 : Figure S2** Phylogenetic tree based on mitochondrial genomes protein coding sequences dataset of Apoidea. The tree was generated by Bayesian Inference; the values above the branches show the posterior probability for Bayesian Inference and values below the branches represent bootstrap support values of the Maximum Likelihood analyses.

**Additional file 3 : Figure S3** Phylogenetic tree generated by Maximum Likelihood method, based on the complete mitochondrial genomes dataset of Apoidea.

**Additional file 4 : Figure S4** Phylogenetic tree generated by Maximum Likelihood method, based on mitochondrial genomes protein coding sequences dataset of Apoidea.

**Additional file 5 : Figure S5** Phylogenetic tree based on mitochondrial genomes tRNAs sequences dataset of Apoidea. The tree was generated by Bayesian Inference; the values above the branches show the posterior probability for Bayesian Inference and values below the branches represent bootstrap support values of the Maximum Likelihood analyses.

**Additional file 6 : Figure S6** Phylogenetic tree generated by Maximum Likelihood method, based on mitochondrial genomes tRNAs sequences dataset of Apoidea.

**Additional file 7 : Figure S7** Unrooted phylogenetic trees for core set of genes of the Toll (A) and IMD/JNK pathways. Amino acid sequences were aligned using MAFFT and the tree was generated in an ML approach (1000 replicates). In red are the orthologs of *Frieseomelitta varia*. Gene names are abbreviated. Species names are in three letters acronyms, Acer: *Apis cerana*, Ador: *Apis dorsata*, Aflo: *Apis florea*, Amel: *Apis mellifera*, Bter: *Bombus terrestris*, Bimp: *Bombus impatiens*, Dnov: *Dufourea novaeangliae*, Edil: *Euglossa dilemma*, Emex: *Eufriesea mexicana*, Fvar: *Frieseomelitta varia*, Hlab: *Habropoda laboriosa,* Lalb: *Lasioglossum albipes*, Mrot: *Megachile rotundata*, Mqua: *Melipona quadrifasciata.*

**Additional file 8 : Figure S8** Unrooted phylogenetic trees for core set of genes of the JAK/STAT and RNAi pathways. Amino acid sequences were aligned using MAFFT and the tree was generated in an ML approach (1000 replicates). In red are the orthologs of *Frieseomelitta varia*. Gene names are abbreviated. Species names are in three letters acronyms, Acer: *Apis cerana*, Ador: *Apis dorsata*, Aflo: *Apis florea*, Amel: *Apis mellifera*, Bter: *Bombus terrestris*, Bimp: *Bombus impatiens*, Dnov: *Dufourea novaeangliae*, Edil: *Euglossa dilemma*, Emex: *Eufriesea mexicana*, Fvar: *Frieseomelitta varia*, Hlab: *Habropoda laboriosa,* Lalb: *Lasioglossum albipes*, Mrot: *Megachile rotundata*, Mqua: *Melipona quadrifasciata.*

**Additional file 9 : Table S1** Genome databases used in blastp searches for protein-coding genes included in the gene set for manual curation of their MAKER 2 gene model predictions.

**Additional file 10 : Table S2** Genome databases used in the prediction of non-coding genes in the *F. varia* genome assembly.

**Additional file 11 : Table S3** Mitochondrial genomes used for the Apoidea phylogenetic tree reconstruction. * denotes the species used as outgroups.

**Additional file 12 : Table S4** Results of the manual curation of 533 gene models generated by automatic prediction.

## Data Availability

The *F. varia* genome sequence assembled as Fvar_v1.2 was submitted to the NCBI genome database (GenBank assembly accession: GCA_011392965.1), and the *F. varia* mitochondrial genome assembly was submitted to the NCBI nucleotide database (GenBank accession: CM022150.1). For detailed documentation on assembly and evaluation procedures, including scripts, see https://github.com/dgpinheiro/fvaria.

## Abbreviations

COX2: Cyclooxygenase 2
EC: Enzyme commission
DNMT: DNA methyltransferase
GO: Gene Ontology
HPTML: Histone post-translational modification
JH: Juvenile hormone
MRJP: Major Royal Jelly Protein
mya: million years ago
OBP9: Odorant binding protein 9
QTL: Quantitative trait locus
RAxML: Random axelerated maximum likelihood
TF: transcription factor
