# Supplementary figures and images for: The nuclear and mitochondrial genomes of Frieseomelitta varia – a highly eusocial stingless bee (Meliponini) with a permanently sterile worker caste
Source: BMC Genomics. 2020 Jun 3;21:386. doi: 10.1186/s12864-020-06784-8 (PMC7268684; doi:10.1186/s12864-020-06784-8)

## Slide 1
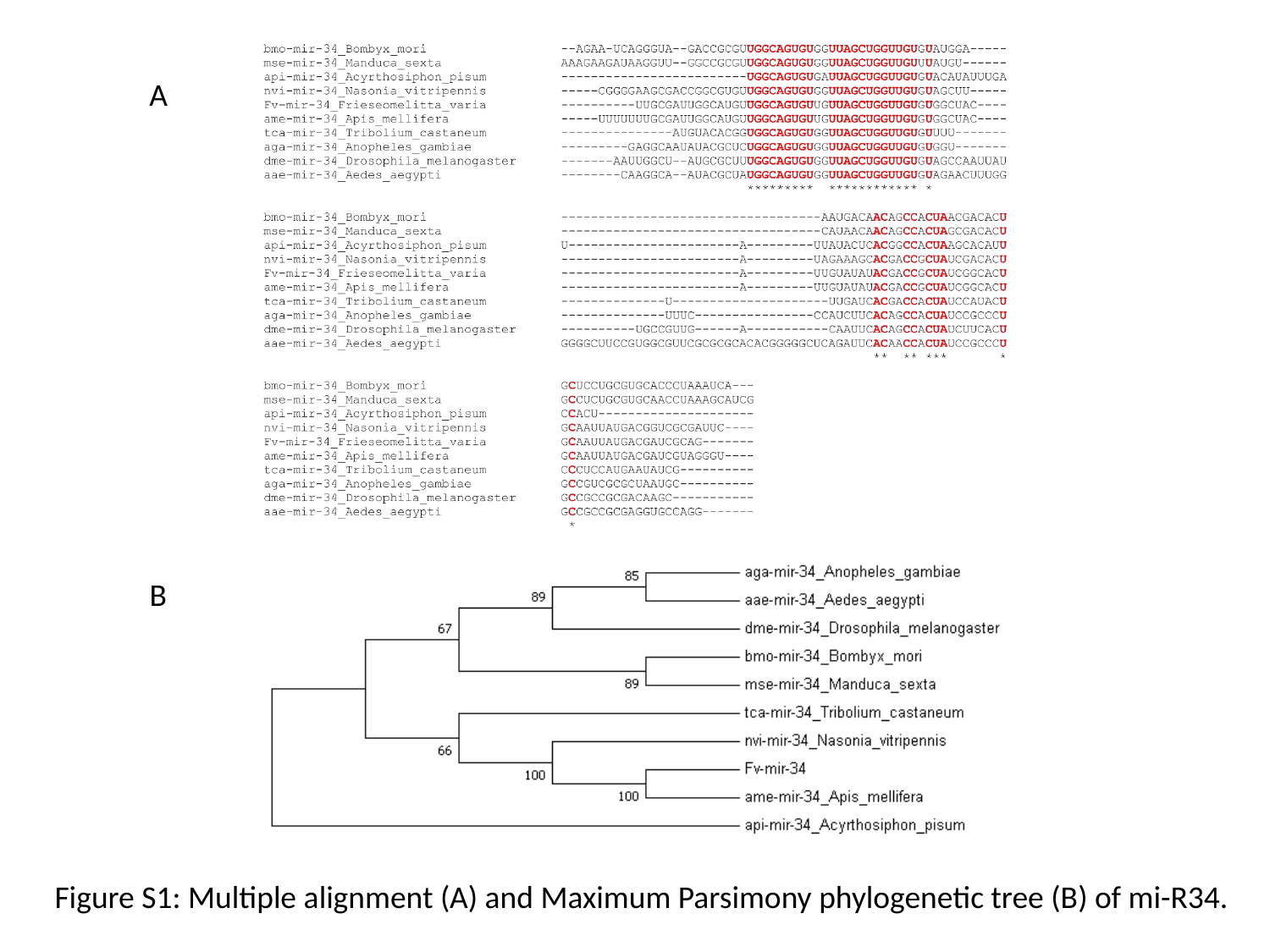

A
B
Figure S1: Multiple alignment (A) and Maximum Parsimony phylogenetic tree (B) of mi-R34.

Supplement: Supplementary file 1 — Additional file 1 : Figure S1: Multiple alignment (A) and Maximum Parsimony phylogenetic tree (B) of miR-34. [file 12864_2020_6784_MOESM1_ESM.pptx]

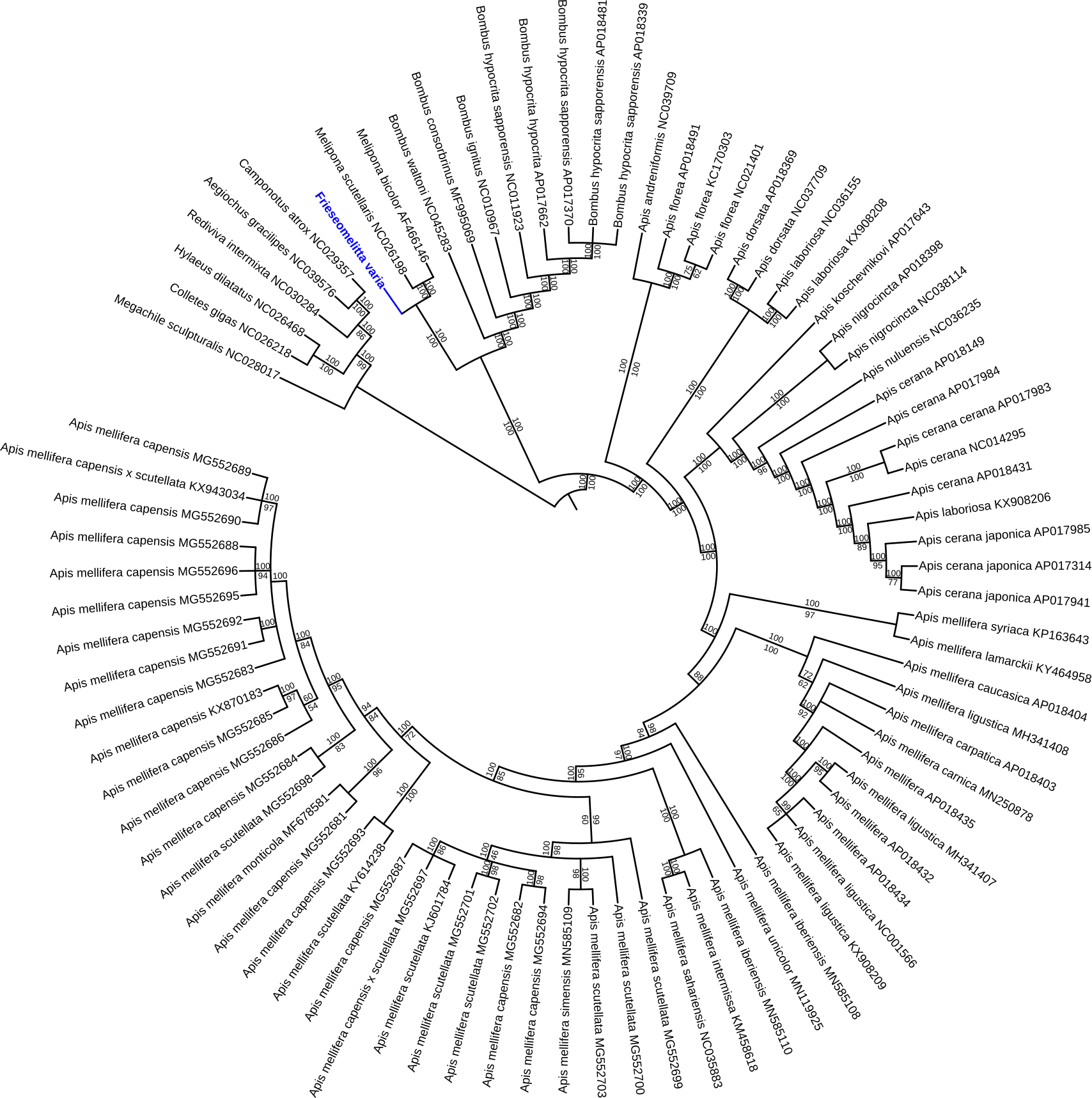

Supplement: Supplementary file 2 — Additional file 2 : Figure S2 Phylogenetic tree based on mitochondrial genomes protein coding sequences dataset of Apoidea. The tree was generated by Bayesian Inference; the values above the branches show the posterior probability for Bayesian Inference and values below the branches represent bootstrap support values of the Maximum Likelihood analyses. [file 12864_2020_6784_MOESM2_ESM.png]

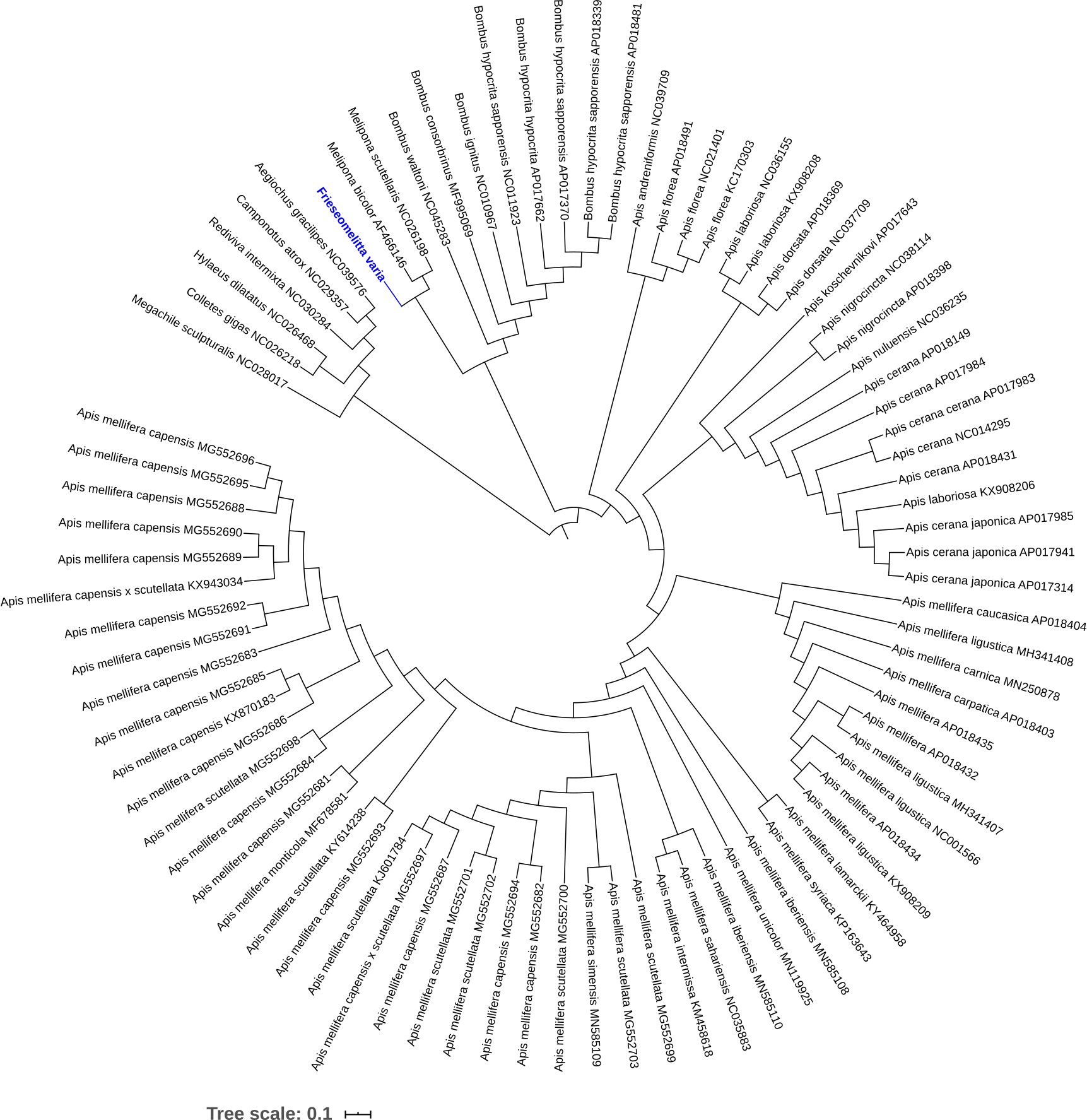

Supplement: Supplementary file 3 — Additional file 3 : Figure S3 Phylogenetic tree generated by Maximum Likelihood method, based on the complete mitochondrial genomes dataset of Apoidea. [file 12864_2020_6784_MOESM3_ESM.png]

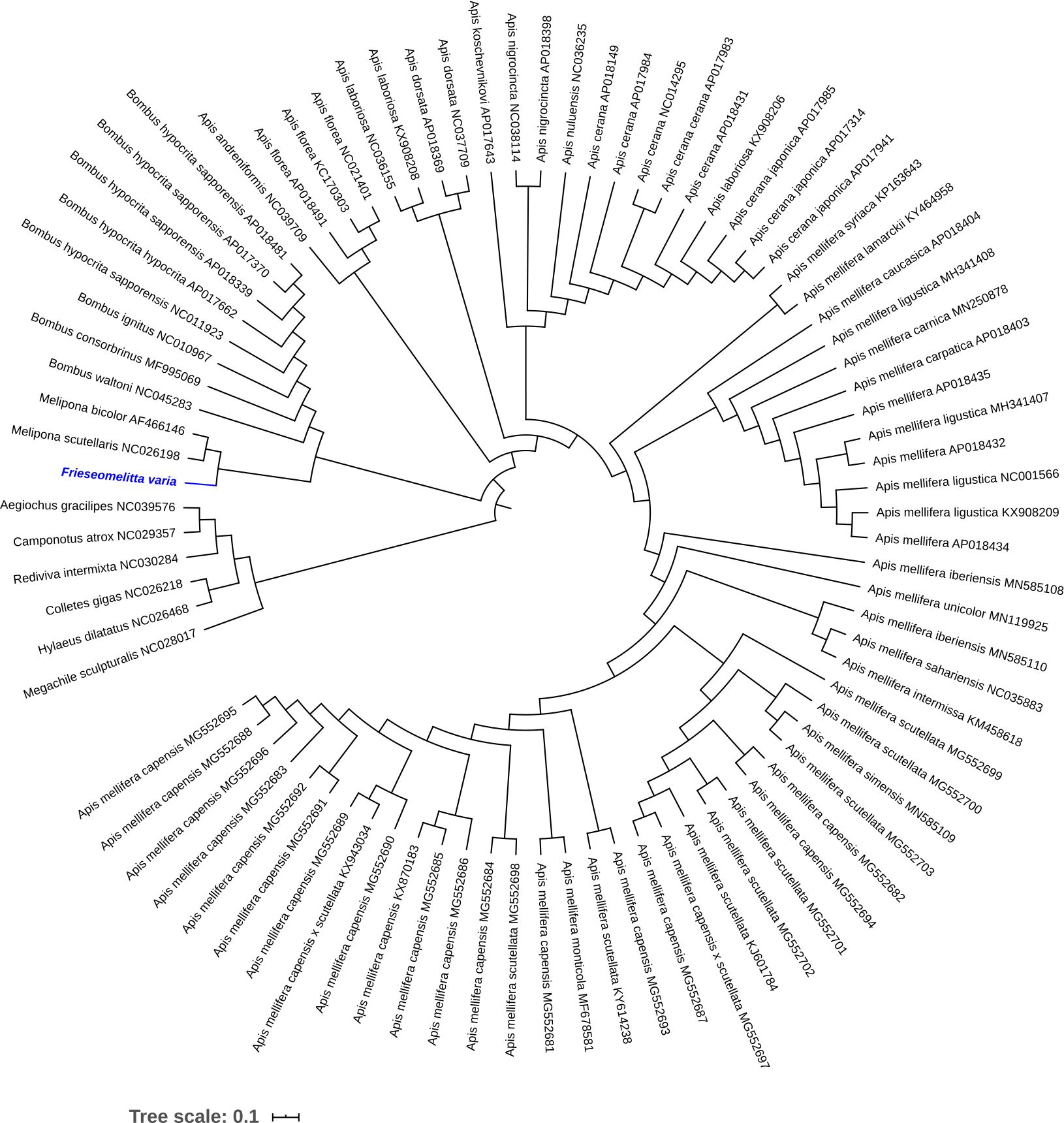

Supplement: Supplementary file 4 — Additional file 4 : Figure S4 Phylogenetic tree generated by Maximum Likelihood method, based on mitochondrial genomes protein coding sequences dataset of Apoidea. [file 12864_2020_6784_MOESM4_ESM.png]

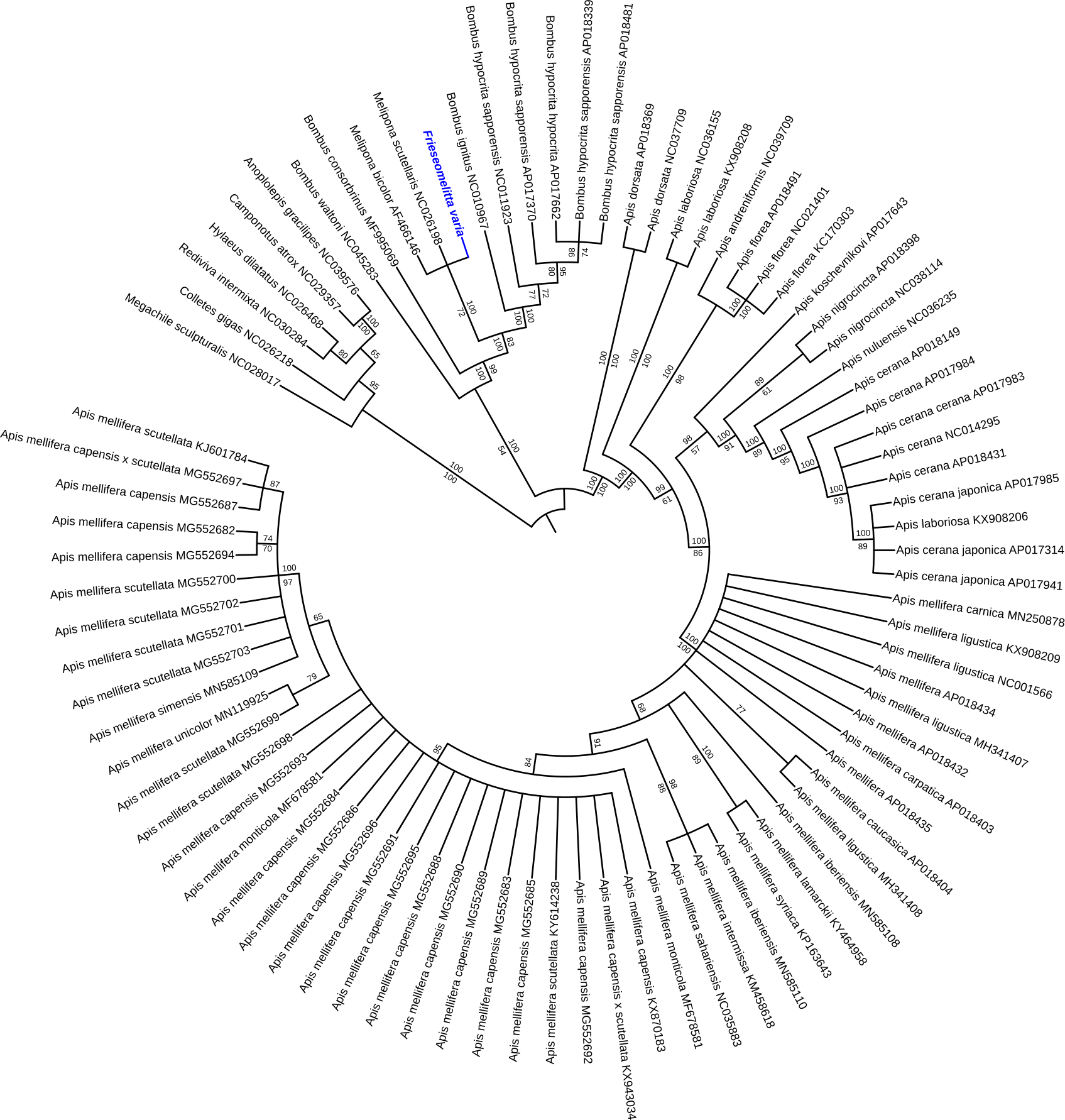

Supplement: Supplementary file 5 — Additional file 5 : Figure S5 Phylogenetic tree based on mitochondrial genomes tRNAs sequences dataset of Apoidea. The tree was generated by Bayesian Inference; the values above the branches show the posterior probability for Bayesian Inference and values below the branches represent bootstrap support values of the Maximum Likelihood analyses. [file 12864_2020_6784_MOESM5_ESM.png]

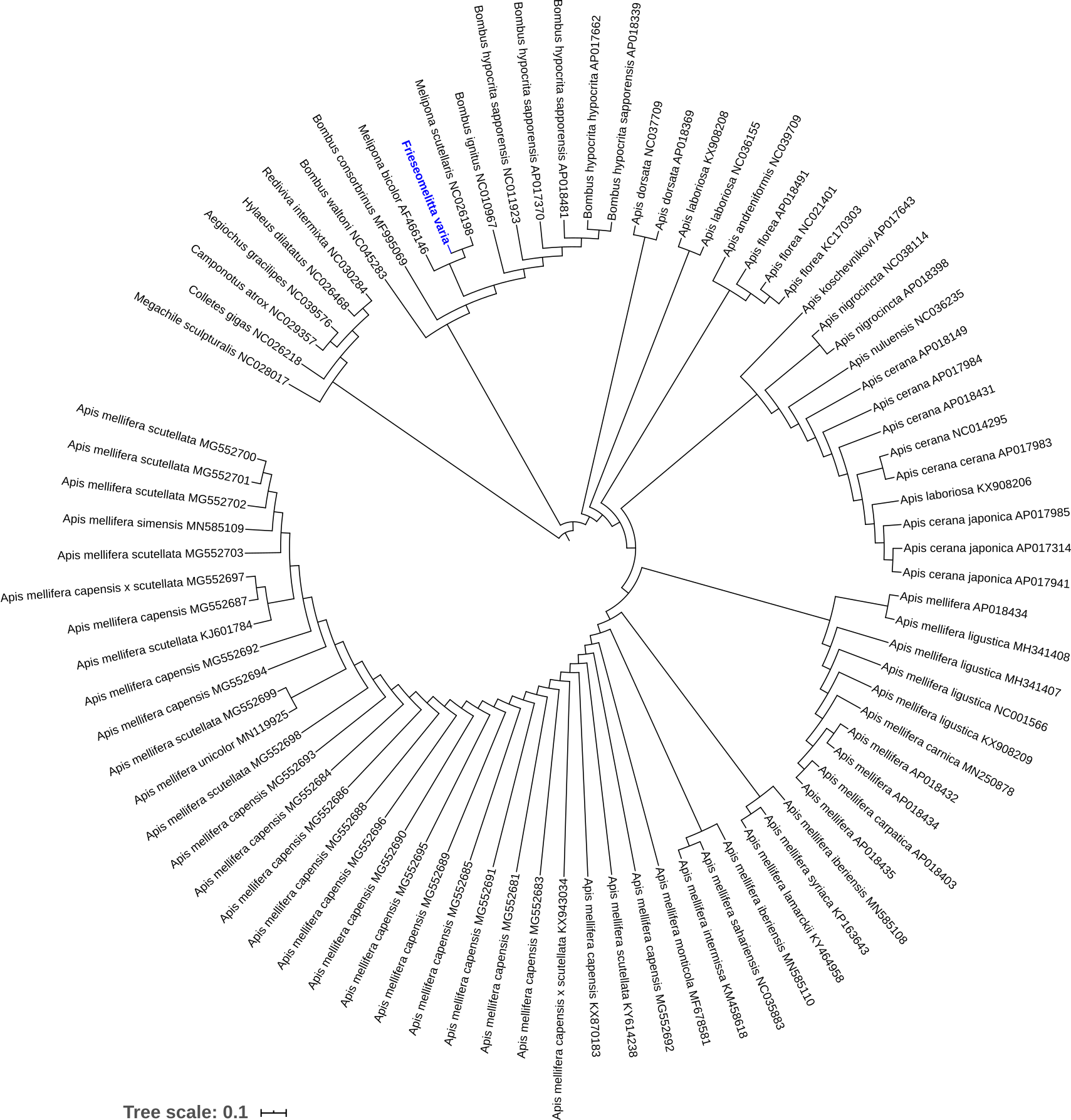

Supplement: Supplementary file 6 — Additional file 6 : Figure S6 Phylogenetic tree generated by Maximum Likelihood method, based on mitochondrial genomes tRNAs sequences dataset of Apoidea. [file 12864_2020_6784_MOESM6_ESM.png]
