# Supplementary material for: The nuclear and mitochondrial genomes of Frieseomelitta varia – a highly eusocial stingless bee (Meliponini) with a permanently sterile worker caste
Source: BMC Genomics. 2020 Jun 3;21:386. doi: 10.1186/s12864-020-06784-8 (PMC7268684; doi:10.1186/s12864-020-06784-8)
Supplement: Supplementary file 8 — Additional file 8 : Figure S8 Unrooted phylogenetic trees for core set of genes of the JAK/STAT and RNAi pathways. Amino acid sequences were aligned using MAFFT and the tree was generated in an ML approach (1000 replicates). In red are the orthologs of Frieseomelitta varia. Gene names are abbreviated. Species names are in three letters acronyms, Acer: Apis cerana, Ador: Apis dorsata, Aflo: Apis florea, Amel: Apis mellifera, Bter: Bombus terrestris, Bimp: Bombus impatiens, Dnov: Dufourea novaeangliae, Edil: Euglossa dilemma, Emex: Eufriesea mexicana, Fvar: Frieseomelitta varia, Hlab: Habropoda laboriosa, Lalb: Lasioglossum albipes, Mrot: Megachile rotundata, Mqua: Melipona quadrifasciata. [file 12864_2020_6784_MOESM8_ESM.pptx]

## Slide 1
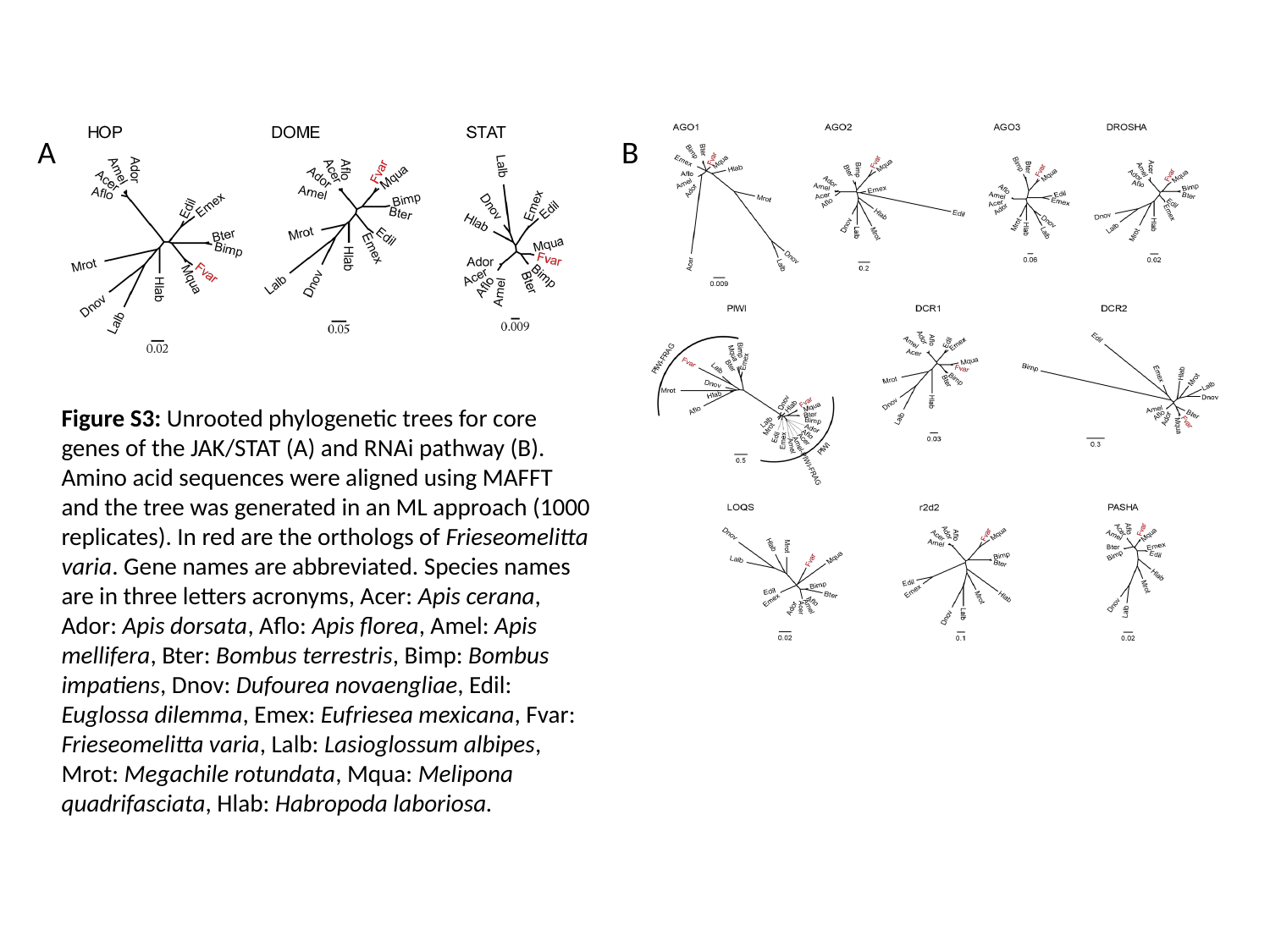

A
B
Figure S3: Unrooted phylogenetic trees for core genes of the JAK/STAT (A) and RNAi pathway (B). Amino acid sequences were aligned using MAFFT and the tree was generated in an ML approach (1000 replicates). In red are the orthologs of Frieseomelitta varia. Gene names are abbreviated. Species names are in three letters acronyms, Acer: Apis cerana, Ador: Apis dorsata, Aflo: Apis florea, Amel: Apis mellifera, Bter: Bombus terrestris, Bimp: Bombus impatiens, Dnov: Dufourea novaengliae, Edil: Euglossa dilemma, Emex: Eufriesea mexicana, Fvar: Frieseomelitta varia, Lalb: Lasioglossum albipes, Mrot: Megachile rotundata, Mqua: Melipona quadrifasciata, Hlab: Habropoda laboriosa.
