# Supplementary material for: The nuclear and mitochondrial genomes of Frieseomelitta varia – a highly eusocial stingless bee (Meliponini) with a permanently sterile worker caste
Source: BMC Genomics. 2020 Jun 3;21:386. doi: 10.1186/s12864-020-06784-8 (PMC7268684; doi:10.1186/s12864-020-06784-8)
Supplement: Supplementary file 9 — Additional file 9 : Table S1 Genome databases used in blastp searches for protein-coding genes included in the gene set for manual curation of their MAKER 2 gene model predictions. [file 12864_2020_6784_MOESM9_ESM.docx]

**Table S1 -** Genome databases used in blastp searches for protein-coding genes included in the gene set for manual curation of their MAKER 2 gene model predictions.

| **Species** | **Symbol** | **Database** | **Version** | **Reference** |
| --- | --- | --- | --- | --- |
| *Apis dorsata* | Ador | NCBI | RefSeq-Release 100 | 1 |
| *Apis florea* | Aflo | NCBI | RefSeq-Release 100 | 1 |
| *Apis mellifera* | Amel | HGDB | amel_OGSv3.2 | 2 |
| *Bombus impatiens* | Bimp | HGDB | Bombus_impatiens_v1.0 | 3 |
|  |  |  | Bombus_impatiens_v1.2 | 4 |
| *Bombus terrestris* | Bter | HGDB | Bombus_terrestris_v1.0 | 3 |
|  |  |  | Bombus_terrestris_v1.3 | 4 |
| *Dufourea novaeangliae* | Dnov | HGDB | Dufourea_novaeangliae_v1.1 | 4 |
| *Eufriesea mexicana* | Emex | HGDB | Eufriesea_mexicana_v1.1 | 4 |
| *Euglossa dilemma* | Edil | HGDB | Edil_OGSv1.0 | 5 |
| *Habropoda laboriosa* | Hlab | HGDB | hsal_OGSv3.3 | 4 |
| *Lasioglossum albipes* | Lalb | HGDB | lalb_OGSv5.42 | 6 |
| *Megachile rotundata* | Mrot | HGDB | Megachile_rotundatav_v1.1 | 4 |
| *Melipona quadrifasciata* | Mqua | HGDB | Melipona_quadrifasciata_v1.1 | 4 |

**References**

[1] O'Leary NA, Wright MW, Brister JR, Ciufo S, Haddad D, McVeigh R, et al. Reference sequence (RefSeq) database at NCBI: current status, taxonomic expansion, and functional annotation. Nucleic Acids Res. 2016;44, D733– D745.

[2] [Elsik CG](https://www.ncbi.nlm.nih.gov/pubmed/?term=Elsik%20CG%5BAuthor%5D&cauthor=true&cauthor_uid=24479613), [Worley KC](https://www.ncbi.nlm.nih.gov/pubmed/?term=Worley%20KC%5BAuthor%5D&cauthor=true&cauthor_uid=24479613), [Bennett AK](https://www.ncbi.nlm.nih.gov/pubmed/?term=Bennett%20AK%5BAuthor%5D&cauthor=true&cauthor_uid=24479613), [Beye M](https://www.ncbi.nlm.nih.gov/pubmed/?term=Beye%20M%5BAuthor%5D&cauthor=true&cauthor_uid=24479613), [Camara F](https://www.ncbi.nlm.nih.gov/pubmed/?term=Camara%20F%5BAuthor%5D&cauthor=true&cauthor_uid=24479613), Childers, et al. (2014) Finding the missing honey bee genes: lessons learned from a genome upgrade.

[BMC Genomics.](https://www.ncbi.nlm.nih.gov/pubmed/24479613) 2014;15:86

[3] Sadd BM, Barribeau SM, Bloch G, de Graaf DC, Dearden P, Elsik CG, et al. (2015) [The genomes of two key bumblebee species with primitive eusocial organization.](https://www.ncbi.nlm.nih.gov/pubmed/25908251) Genome Biol. 2015;16:76.

[4] Kapheim KM, Pan H, Li C, Salzberg SL, Puiu D, Magoc T. Genomic signatures of evolutionary transitions from solitary to group living. Science. 2015;348:1139-43.

[5] [Brand P](https://www.ncbi.nlm.nih.gov/pubmed/?term=Brand%20P%5BAuthor%5D&cauthor=true&cauthor_uid=28701376), [Saleh N](https://www.ncbi.nlm.nih.gov/pubmed/?term=Saleh%20N%5BAuthor%5D&cauthor=true&cauthor_uid=28701376), [Pan H](https://www.ncbi.nlm.nih.gov/pubmed/?term=Pan%20H%5BAuthor%5D&cauthor=true&cauthor_uid=28701376), [Li C](https://www.ncbi.nlm.nih.gov/pubmed/?term=Li%20C%5BAuthor%5D&cauthor=true&cauthor_uid=28701376), [Kapheim KM](https://www.ncbi.nlm.nih.gov/pubmed/?term=Kapheim%20KM%5BAuthor%5D&cauthor=true&cauthor_uid=28701376), [Ramírez SR](https://www.ncbi.nlm.nih.gov/pubmed/?term=Ram%C3%ADrez%20SR%5BAuthor%5D&cauthor=true&cauthor_uid=28701376). The nuclear and mitochondrial genomes of the facultatively eusocial orchid bee *Euglossa dilemma*. [G3 (Bethesda)](https://www.ncbi.nlm.nih.gov/pubmed/28701376). 2017;7:2891-2898.

[6] Kocher SD, Li C, Yang W, Tan H, Yi SV. [The draft genome of a socially polymorphic halictid bee, *Lasioglossum albipes*.](https://www.ncbi.nlm.nih.gov/pubmed/24359881) Genome Biol. 2013; 14:R142.
