# Supplementary material for: The nuclear and mitochondrial genomes of Frieseomelitta varia – a highly eusocial stingless bee (Meliponini) with a permanently sterile worker caste
Source: BMC Genomics. 2020 Jun 3;21:386. doi: 10.1186/s12864-020-06784-8 (PMC7268684; doi:10.1186/s12864-020-06784-8)
Supplement: Supplementary file 10 — Additional file 10 : Table S2 Genome databases used in the prediction of non-coding genes in the F. varia genome assembly. [file 12864_2020_6784_MOESM10_ESM.docx]

**Table S2 -** Genome databases used in the prediction of non-coding genes in the *F. varia* genome assembly.

| **Species** | **Genome version (ENSEMBL)** |
| --- | --- |
| *Anopheles gambiae* | AgamP4 |
| *Apis mellifera* | Amel_4.5 |
| *Atta cephalotes* | Attacep1.0 |
| *Bombus impatiens* | BIMP_2.0 |
| *Bombyx mori* | ASM15162v1 |
| *Drosophila melanogaster* | BDGP6 |
| *Nasonia vitripennis* | Nvit_2.1 |
| *Solenopsis invicta* | Si_gnG |
