# Supplementary material for: The nuclear and mitochondrial genomes of Frieseomelitta varia – a highly eusocial stingless bee (Meliponini) with a permanently sterile worker caste
Source: BMC Genomics. 2020 Jun 3;21:386. doi: 10.1186/s12864-020-06784-8 (PMC7268684; doi:10.1186/s12864-020-06784-8)
Supplement: Supplementary file 11 — Additional file 11 : Table S3 Mitochondrial genomes used for the Apoidea phylogenetic tree reconstruction. * denotes the species used as outgroups. [file 12864_2020_6784_MOESM11_ESM.docx]

**Table S3 -** Mitochondrial genomes used for the Apoidea phylogenetic tree reconstruction.

| **Accession number** | **Organism name** | **Description** | **Sequence length (bp)** |
| --- | --- | --- | --- |
| NC_039709.1 | *Apis andreniformis* | Apis andreniformis mitochondrial DNA, complete genome | 16,694 |
| AP017983.2 | *Apis cerana cerana* | Apis cerana cerana mitochondrial DNA, complete sequence | 15,460 |
| AP017941.1 | *Apis cerana japonica* | Apis cerana japonica mitochondrial DNA, complete genome | 15,788 |
| AP017314.1 | *Apis cerana japonica* | Apis cerana japonica mitochondrial DNA, complete genome | 15,917 |
| AP017985.1 | *Apis cerana japonica* | Apis cerana japonica mitochondrial DNA, complete sequence | 15,339 |
| AP018431.1 | *Apis cerana* | Apis cerana mitochondrial DNA, complete genome | 15,925 |
| AP018149.1 | *Apis cerana* | Apis cerana mitochondrial DNA, complete genome | 15,884 |
| AP017984.2 | *Apis cerana* | Apis cerana mitochondrial DNA, complete sequence | 15,376 |
| NC_014295.1 | *Apis cerana* | Apis cerana mitochondrion, complete genome | 15,895 |
| KX908206.1 | *Apis cerana* | Apis cerana mitochondrion, complete genome | 15,904 |
| AP018369.1 | *Apis dorsata* | Apis dorsata mitochondrial DNA, complete genome | 15,279 |
| NC_037709.1 | *Apis dorsata* | Apis dorsata mitochondrion, complete genome | 15,892 |
| AP018491.1 | *Apis florea* | Apis florea mitochondrial DNA, complete genome | 17,693 |
| NC_021401.1 | *Apis florea* | Apis florea mitochondrion, complete genome | 17,694 |
| KC170303.1 | *Apis florea* | Apis florea mitochondrion, complete genome | 15,993 |
| AP017643.1 | *Apis koschevnikovi* | Apis koschevnikovi mitochondrial DNA, complete sequence | 15,278 |
| NC_036155.2 | *Apis laboriosa* | Apis laboriosa mitochondrial DNA, complete sequence | 15,510 |
| KX908208.1 | *Apis laboriosa* | Apis laboriosa mitochondrion, complete genome | 15,621 |
| MN585109.1 | *Apis mellifera adansonii* | Apis mellifera adansonii voucher 1284 mitochondrion, complete genome | 16,466 |
| KX870183.1 | *Apis mellifera capensis* | Apis mellifera capensis mitochondrion, complete genome | 16,470 |
| MG552681.1 | *Apis mellifera capensis* | Apis mellifera capensis voucher BD mitochondrion, complete genome | 16,467 |
| MG552682.1 | *Apis mellifera capensis* | Apis mellifera capensis voucher CD mitochondrion, complete genome | 16,447 |
| MG552683.1 | *Apis mellifera capensis* | Apis mellifera capensis voucher CT mitochondrion, complete genome | 16,442 |
| MG552684.1 | *Apis mellifera capensis* | Apis mellifera capensis voucher GE mitochondrion, complete genome | 16,380 |
| MG552685.1 | *Apis mellifera capensis* | Apis mellifera capensis voucher GT mitochondrion, complete genome | 16,428 |
| MG552686.1 | *Apis mellifera capensis* | Apis mellifera capensis voucher LA mitochondrion, complete genome | 16,473 |
| MG552687.1 | *Apis mellifera capensis* | Apis mellifera capensis voucher LB mitochondrion, complete genome | 16,468 |
| MG552688.1 | *Apis mellifera capensis* | Apis mellifera capensis voucher MB mitochondrion, complete genome | 16,453 |
| MG552689.1 | *Apis mellifera capensis* | Apis mellifera capensis voucher MF mitochondrion, complete genome | 16,463 |
| MG552690.1 | *Apis mellifera capensis* | Apis mellifera capensis voucher PB mitochondrion, complete genome | 16,434 |
| MG552691.1 | *Apis mellifera capensis* | Apis mellifera capensis voucher PE mitochondrion, complete genome | 16,515 |
| MG552692.1 | *Apis mellifera capensis* | Apis mellifera capensis voucher RD mitochondrion, complete genome | 16,435 |
| MG552693.1 | *Apis mellifera capensis* | Apis mellifera capensis voucher SF mitochondrion, complete genome | 16,467 |
| MG552694.1 | *Apis mellifera capensis* | Apis mellifera capensis voucher ST mitochondrion, complete genome | 16,344 |
| MG552695.1 | *Apis mellifera capensis* | Apis mellifera capensis voucher SW mitochondrion, complete genome | 16,439 |
| MG552696.1 | *Apis mellifera capensis* | Apis mellifera capensis voucher WD mitochondrion, complete genome | 16,459 |
| MG552697.1 | *Apis mellifera capensis* x *Apis mellifera scutellata* | Apis mellifera capensis x Apis mellifera scutellata voucher KL mitochondrion, complete genome | 16,456 |
| MN250878.1 | *Apis mellifera carnica* | Apis mellifera carnica mitochondrion, complete genome | 16,358 |
| AP018403.1 | *Apis mellifera carpatica* | Apis mellifera carpatica mitochondrial DNA, complete genome | 16,336 |
| AP018404.1 | *Apis mellifera caucasica* | Apis mellifera caucasica mitochondrial DNA, complete genome | 16,341 |
| MN585110.1 | *Apis mellifera iberiensis* | Apis mellifera iberiensis voucher 1964 mitochondrion, complete genome | 16,560 |
| KM458618.1 | *Apis mellifera intermissa* | Apis mellifera intermissa mitochondrion, complete genome | 16,336 |
| KY464958.1 | *Apis mellifera lamarckii* | Apis mellifera lamarckii voucher 1842 mitochondrion, complete genome | 16,589 |
| NC_001566.1 | *Apis mellifera ligustica* | Apis mellifera ligustica mitochondrion, complete genome | 16,343 |
| KX908209.1 | *Apis mellifera ligustica* | Apis mellifera ligustica mitochondrion, complete genome | 16,465 |
| MH341407.1 | *Apis mellifera ligustica* | Apis mellifera ligustica voucher CNU7293 mitochondrion, complete ,genome | 16,449 |
| MH341408.1 | *Apis mellifera ligustica* | Apis mellifera ligustica voucher CNU7294 mitochondrion, complete genome | 16,426 |
| AP018434.1 | *Apis mellifera* | Apis mellifera mitochondrial DNA, complete genome, strain: Blackbee | 16,336 |
| AP018432.1 | *Apis mellifera* | Apis mellifera mitochondrial DNA, complete genome, strain: buckfast | 16,353 |
| AP018435.1 | *Apis mellifera* | Apis mellifera mitochondrial DNA, complete genome, strain: Italian | 16,349 |
| MF678581.1 | *Apis mellifera monticola* | Apis mellifera monticola voucher 1626 mitochondrion, complete genome | 16,343 |
| NC_035883.1 | *Apis mellifera sahariensis* | Apis mellifera sahariensis mitochondrion, complete genome | 16,569 |
| KJ601784.1 | *Apis mellifera scutellata* | Apis mellifera scutellata mitochondrion, complete genome | 16,411 |
| KY614238.1 | *Apis mellifera scutellata* | Apis mellifera scutellata voucher 1982 mitochondrion, complete genome | 16,288 |
| MG552698.1 | *Apis mellifera scutellata* | Apis mellifera scutellata voucher BL mitochondrion, complete genome | 16,479 |
| MG552699.1 | *Apis mellifera scutellata* | Apis mellifera scutellata voucher KR mitochondrion, complete genome | 16,454 |
| MG552700.1 | *Apis mellifera scutellata* | Apis mellifera scutellata voucher PT mitochondrion, complete genome | 16,462 |
| MG552701.1 | *Apis mellifera scutellata* | Apis mellifera scutellata voucher SP mitochondrion, complete genome | 16,450 |
| MG552702.1 | *Apis mellifera scutellata* | Apis mellifera scutellata voucher UP mitochondrion, complete genome | 16,364 |
| MG552703.1 | *Apis mellifera scutellata* | Apis mellifera scutellata voucher VR mitochondrion, complete genome | 16,339 |
| KX943034.1 | *Apis mellifera scutellata x Apis mellifera capensis* | Apis mellifera scutellata x Apis mellifera capensis mitochondrion, complete genome | 16,340 |
| MN585108.1 | *Apis mellifera simensis* | Apis mellifera simensis voucher 2721 mitochondrion, complete genome | 16,523 |
| KP163643.1 | *Apis mellifera syriaca* | Apis mellifera syriaca mitochondrion, complete genome | 15,428 |
| MN119925.1 | *Apis mellifera unicolor* | Apis mellifera unicolor mitochondrion, complete genome | 16,373 |
| AP018398.1 | *Apis nigrocincta* | Apis nigrocincta mitochondrial DNA, complete genome | 15,516 |
| NC_038114.1 | *Apis nigrocincta* | Apis nigrocincta mitochondrion, complete genome | 15,855 |
| NC_036235.1 | *Apis nuluensis* | Apis nuluensis voucher 1929 mitochondrion, complete genome | 15,843 |
| MF995069.1 | *Bombus consobrinus* | Bombus consobrinus mitochondrion, complete genome | 17,966 |
| AP017662.1 | *Bombus hypocrita hypocrita* | Bombus hypocrita hypocrita mitochondrial DNA, complete sequence | 15,795 |
| AP018339.1 | *Bombus hypocrita sapporensis* | Bombus hypocrita sapporensis mitochondrial DNA, complete genome | 16,133 |
| AP018481.1 | *Bombus hypocrita sapporensis* | Bombus hypocrita sapporensis mitochondrial DNA, complete genome, country: Japan:Otofuke | 15,835 |
| AP017370.2 | *Bombus hypocrita sapporensis* | Bombus hypocrita sapporensis mitochondrial DNA, complete sequence | 15,826 |
| NC_011923.1 | *Bombus hypocrita sapporensis* | Bombus hypocrita sapporensis mitochondrion, complete genome | 15,468 |
| NC_010967.1 | *Bombus ignitus* | Bombus ignitus mitochondrion, complete genome | 16,434 |
| NC_045283.1 | *Bombus waltoni* | Bombus waltoni mitochondrion, complete genome | 19,349 |
| AF466146.2 | *Melipona bicolor* | Melipona bicolor mitochondrion, complete genome | 15,001 |
| NC_026198.1 | *Melipona scutellaris* | Melipona scutellaris mitochondrion, complete genome | 14,862 |
| NC_039576.1 | *Anoplolepis gracilipes** | Anoplolepis gracilipes voucher Ano062 mitochondrion, complete genome | 16,943 |
| NC_030284.1 | *Rediviva intermixta** | Rediviva intermixta mitochondrion, complete genome | 16,875 |
| NC_029357.1 | *Camponotus atrox** | Camponotus atrox mitochondrion, complete genome | 16,540 |
| NC_028017.1 | *Megachile sculpturalis** | Megachile sculpturalis mitochondrion, complete genome | 16,581 |
| NC_026468.1 | *Hylaeus dilatatus** | Hylaeus dilatatus voucher R0131103811 mitochondrion, complete genome | 15,475 |
| NC_026218.1 | *Colletes gigas** | Colletes gigas mitochondrion, complete genome | 15,885 |

* denotes the species used as outgroups.
