# Supplementary material for: The nuclear and mitochondrial genomes of Frieseomelitta varia – a highly eusocial stingless bee (Meliponini) with a permanently sterile worker caste
Source: BMC Genomics. 2020 Jun 3;21:386. doi: 10.1186/s12864-020-06784-8 (PMC7268684; doi:10.1186/s12864-020-06784-8)
Supplement: Supplementary file 12 — Additional file 12 : Table S4 Results of the manual curation of 533 gene models generated by automatic prediction. [file 12864_2020_6784_MOESM12_ESM.docx]

**Table S4 -** Results of the manual curation of 533 gene models generated by automatic prediction.

| **Manual curation result** | **Gene number** |
| --- | --- |
| Automatic prediction confirmed as correct | 241 |
| Error in exon number or position | 109 |
| Wrong prediction of first exon or lack of methionine start codon | 65 |
| Wrong size of individual exons | 57 |
| Two or more or two or more genes with the same identifier | 27 |
| Gene split on different scaffolds, gene was only found manually | 18 |
| Problem with intron/exon boundary | 8 |
| Evidence for sequencing error, with nucleotides lacking | 7 |
| Evidence that UTR was predicted as containing exon | 1 |
